# Probiotic‐Derived Vesicles Rectally Delivered via Thermo‐Gelling Copolymer Promote Mucosal Healing and Reduce Inflammation in Ulcerative Colitis

**DOI:** 10.1002/advs.76585

**Published:** 2026-07-17

**Authors:** Ayushi Mairal, Ubaid Tariq, Shreya Mehrotra, Saravanan Matheshwaran, Jan Marsal, Ashok Kumar

**Affiliations:** ^1^ Department of Biological Sciences and Bioengineering Indian Institute of Technology Kanpur Kanpur UP India; ^2^ Centre For Environmental Science and Engineering Indian Institute of Technology Kanpur Kanpur UP India; ^3^ The Mehta Family Centre For Engineering in Medicine Indian Institute of Technology Kanpur Kanpur UP India; ^4^ Department of Clinical Sciences Lund University and Skåne University Hospital Lund Sweden; ^5^ Centre of Excellence for Materials in Medicine Gangwal School of Medical Sciences and Technology Indian Institute of Technology Kanpur Kanpur UP India; ^6^ Centre For Nanosciences Indian Institute of Technology Kanpur Kanpur UP India

**Keywords:** extracellular vesicles, mucosal healing, thermo‐responsive delivery, ulcerative colitis

## Abstract

Ulcerative colitis remains challenging to treat due to the need for localized control of inflammation, restoration of mucosal integrity, and avoidance of systemic toxicity. Here, we report a rectally administered, sprayable, thermo‐responsive hyaluronic acid‐based copolymer platform for the localized co‐delivery of probiotic‐derived extracellular vesicles (ProEVs) and 5‐aminosalicylic acid. This system enables enhanced retention and sustained presentation of therapeutics at inflamed colonic sites. The combined formulation demonstrates improved therapeutic efficacy compared to individual treatments, promoting mucosal recovery and intestinal homeostasis. Mechanistically, the platform modulates inflammatory responses, supports epithelial barrier function, and contributes to microbiota rebalancing. Notably, extracellular vesicles provide a more effective and consistent therapeutic modality compared to live probiotics. Importantly, the formulation is designed for clinical translation, offering localized administration, minimal toxicity, and stability upon lyophilization, enabling off‐the‐shelf use. These findings highlight the potential of integrating extracellular vesicles with biomaterial‐based delivery systems as a multifunctional therapeutic strategy for inflammatory bowel disease.

## Graphical abstract

A thermo‐responsive rectal delivery platform integrating probiotic‐derived extracellular vesicles within a hyaluronic acid‐based copolymer enables localized and sustained therapy for ulcerative colitis. The formulation effectively suppresses inflammation, promotes macrophage polarization toward a regenerative phenotype, restores epithelial barrier integrity, and enhances mucosal healing and gut microbiota recovery, highlighting its potential as a clinically translatable cell‐free therapeutic strategy for inflammatory bowel disease.

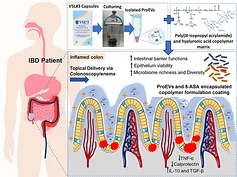

## 1 Introduction

Inflammatory bowel disease (IBD) is a chronic, relapsing disorder of the gastrointestinal tract characterized by symptoms such as abdominal pain [1], diarrhea [2], rectal bleeding [3], and weight loss [4]. Its pathogenesis is driven by a complex interplay between genetic predisposition, environmental factors, immune dysregulation, and perturbations in the gut microbiota [5, 6]. With a rapidly rising prevalence in newly industrialized regions, IBD poses substantial public health challenges due to escalating healthcare costs, reduced productivity, and impaired quality of life [7, 8].

While current pharmacological strategies, including amino‐salicylates [9], corticosteroids [10], immunomodulators [11], biologics [12], and Janus kinase (JAK) inhibitors [13], are effective in managing acute inflammation, they are often associated with serious adverse effects such as hepatotoxicity, infections, and malignancies [14, 15, 16]. Additionally, these therapies frequently disrupt the delicate equilibrium of the gut microbiome, potentially exacerbating dysbiosis and triggering disease relapses [17]. Moreover, these therapies do not adequately address key disease‐driving factors such as epithelial barrier dysfunction and microbial dysbiosis, which contribute to disease recurrence and chronicity. Thus, there is a growing need for next‐generation therapeutic approaches that not only suppress inflammation but also restore mucosal integrity and microbial homeostasis.

In this context, probiotics have attracted significant interest for their ability to modulate the gut microbiome, enhance epithelial barrier integrity, and regulate host immune responses. Among them, VSL#3, a well‐characterized multi‐strain formulation comprising *Lactobacillus*, *Bifidobacterium*, and *Streptococcus* species, has demonstrated clinical efficacy in improving barrier function and maintaining remission in IBD [18, 19]. Recent advances in probiotic delivery such as microencapsulation [20], colon‐targeted release systems [21], and engineered bacterial strains [22] aim to enhance therapeutic efficacy. However, the therapeutic potential of live probiotic formulations is often constrained by limited viability, inconsistent colonization, and variability in host responses. Emerging evidence suggests that many of the beneficial effects of probiotics are mediated through their secreted bioactive components rather than the live bacteria themselves. In this regard, extracellular vesicles derived from probiotic strains represent a promising cell‐free alternative, capable of retaining key functional properties while offering improved stability, reproducibility, and translational potential [23, 24]. Secreted by both Gram‐positive and Gram‐negative bacteria, EVs are nanoscale vesicles (20–300 nm) that encapsulate proteins, nucleic acids, lipids, and peptidoglycans capable of mediating host‐microbe communication [25, 26]. EVs can traverse intestinal barriers, interact directly with immune and epithelial cells, and activate signaling cascades that modulate inflammation and tissue repair, making them potent mediators of gut health [24, 27].

However, a major challenge limiting the therapeutic application of EVs is their rapid clearance and insufficient retention at target sites within the gastrointestinal tract. The inflamed colonic environment is characterized by mucus turnover, luminal flow, and heterogeneous lesion distribution, all of which hinder effective localization and sustained activity of therapeutics. Biomaterial‐based delivery systems offer a compelling strategy to overcome these limitations. In particular, thermo‐responsive and mucoadhesive polymeric systems can enable localized, in situ gelation and prolonged retention of therapeutic agents at diseased sites, thereby enhancing their bioavailability and efficacy.

Here, we report the development of a thermo‐responsive hyaluronic acid‐based copolymer platform (PNHA) for the localized delivery of probiotic‐derived extracellular vesicles (ProEVs), isolated from a multi‐strain probiotic formulation VSL#3. This formulation was designed as a therapeutic drug delivery platform for topical application during endoscopy, targeting inflamed colon tissue directly. Hyaluronic acid provides intrinsic mucoadhesive properties and potential interactions with inflamed tissues, while the thermo‐responsive polymer enables in situ gelation at physiological temperatures, facilitating sustained retention within the colonic mucosa [28, 29, 30]. By integrating the biological functionality of ProEVs with the spatiotemporal control afforded by the PNHA matrix, this platform is designed to enhance therapeutic localization and efficacy.

Collectively, this study presents a biomaterial‐assisted EV delivery strategy aimed at addressing key limitations of current IBD therapies by combining immunomodulation, barrier restoration, and microbiota‐directed effects within a single, localized treatment platform. Importantly, the formulation is designed with clinical translation in mind, offering a minimally invasive, localized delivery approach that can be adapted for endoscopic administration. Furthermore, the ability to preserve the formulation via lyophilization supports its potential as an off‐the‐shelf therapeutic system with extended shelf life, facilitating storage, transport, and large‐scale deployment. Such features highlight the promise of this platform as a clinically relevant and scalable solution for the management of IBD.

## 2 Results and Discussion

### 2.1 Development and Physico‐Chemical Characterization of PNHA‐ProEVs Formulation

The comb‐type graft copolymer P(NIPAM*‐co‐*NTBAM)‐HA (PNHA) was synthesized via a two‐step process (Figure 1A and Figure S1A) as previously described [29]. First, an amine‐terminated copolymer, P(NIPAM*‐co‐*NTBAM)‐NH_2_, was obtained by free radical polymerization of NIPAM and NTBAM in a 9:1 molar ratio, using a KPS/AET·HCl redox initiator system. FTIR confirmed successful polymerization and amine termination, with characteristic amide I and II peaks (1654 and 1553 cm^−^^1^), N‐H stretching (3439–3302 cm^−^^1^), and the absence of monomer‐specific peaks (Figure S1B). In the second step, the copolymer was grafted onto HA using EDC/NHS coupling. The final conjugate showed combined spectral features of HA and the copolymer, including COO^−^ stretches (1617 and 1420 cm^−^^1^) and PNIPAM‐related peaks, with a shifted carboxyl peak (∼1621 cm^−^^1^) confirming amide bond formation. To maintain a sol‐to‐gel transition below physiological temperature, the hydrophobic NTBAM unit was introduced to counteract HA‐induced LCST elevation. The final PNHA copolymer exhibited a sharp sol‐gel transition at ∼34°C. This temperature‐responsive gelation of PNHA enables rapid in situ solidification at physiological temperature, ensuring effective retention at the target site. Under simulated intestinal conditions (37°C), PNHA maintained gel integrity and strong adhesion to colonic tissue over extended durations and also demonstrated good applicability under simulated colonoscopic conditions (Figure S2A–E and Movies S1 and S2). Notably, PNHA demonstrated significantly higher retention (57.9%) compared to plain PNIPAM without HA (34.73%) at 72 h, which can be attributed to enhanced muco‐adhesion mediated by hyaluronic acid (Figure S2F,G). This sustained retention, despite gradual degradation, highlights PNHA as a promising platform for prolonged mucosal residence and controlled local drug delivery. Such sustained presence underscores the ability of PNHA to act as a long‐term depot system for controlled drug release in the colonic environment.

**FIGURE 1:**
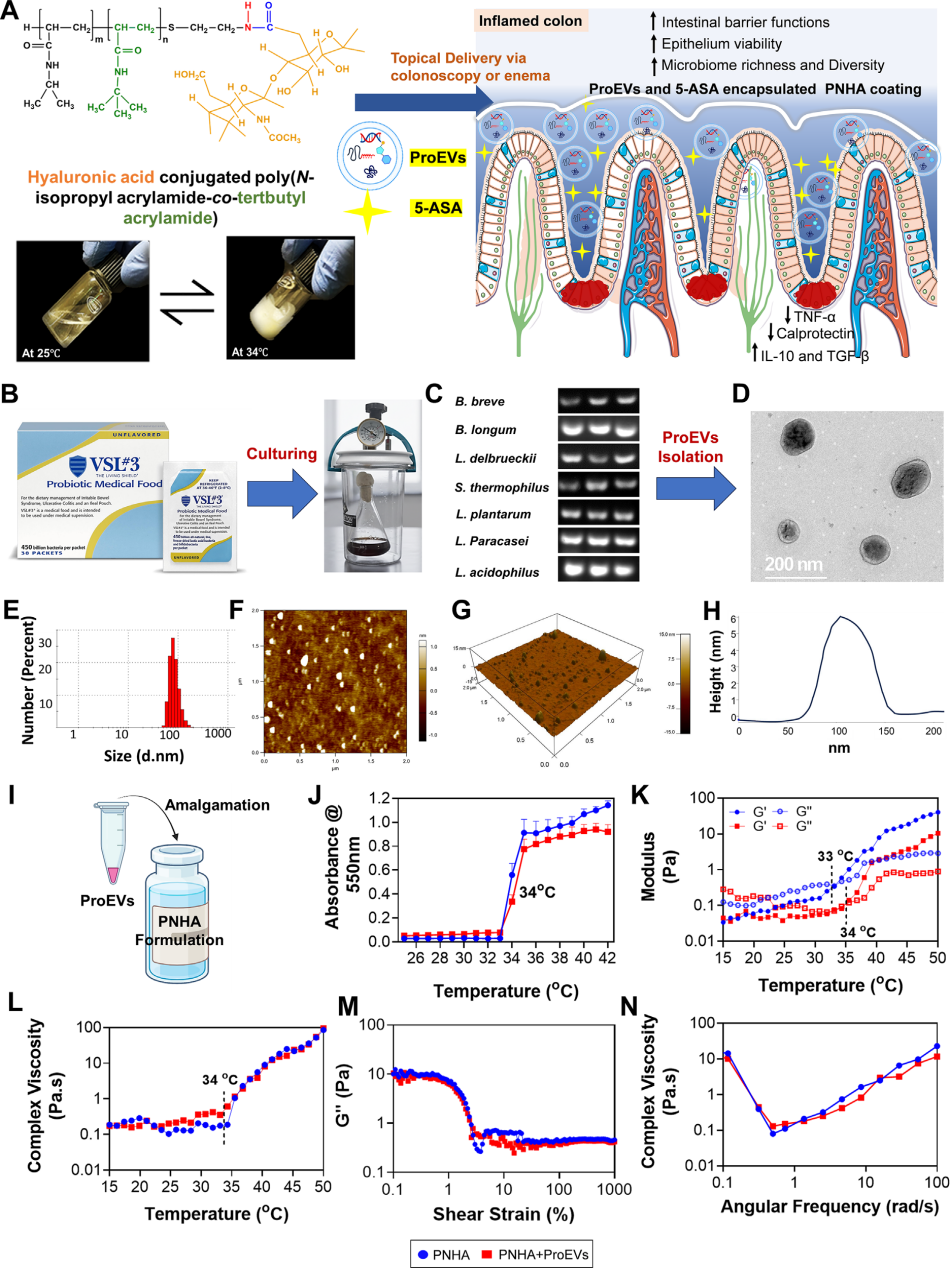
Synthesis and characterization of PNHA‐ProEVs formulation. (A) Chemical structure of PNHA and schematic showing its rectal administration for immunomodulation, epithelial healing, and microbiota restoration in colitis, (B) Schematic of anaerobic culturing of VSL#3 for ProEVs isolation, (C) PCR confirmation of VSL#3 strains via gyrA gene amplification (n = 3), (D) TEM image of ProEVs showing circular nanoscale morphology of vesicle, (E) DLS analysis showing ProEVs with narrow size distribution (∼90–120 nm) (n = 3) (F–H) Two‐ and three‐dimensional atomic force microscopy (AFM) images of ProEVs revealed their characteristic dome‐shaped morphology, with size analysis indicating an average lateral dimension of ∼100 nm and a height of ∼6 nm (n = 3) (I) Graphical illustration of PNHA‐ProEVs formulation preparation, (J) LCST profiles of copolymer with and without ProEVs, indicating stable thermo‐responsiveness (n = 4), (K) Rheological data showing sol‐gel transition and dominance of storage modulus (G′) above LCST (n = 3) (L) Amplitude sweep analysis displaying the change in G″ on increasing the shear strain, which could be useful in determining the threshold limit of linear viscoelasticity (LVE), (M) Temperature‐dependent increase in complex viscosity confirms thermo‐gelling behavior (n = 4), (N) Frequency sweep analysis for determining the stability of viscoelastic polymer formulations PNHA and PNHA‐ProEVs. Data are presented as mean ± s.d.; ^*^ *p* < 0.05, ^**^ *p* < 0.01, ^***^ *p* < 0.001, ^***^ *p* < 0.0001.

To prepare the vesicle component of the system, we cultivated the multi‐strain probiotic VSL#3 under anaerobic conditions using MRS broth to ensure viability of obligate anaerobes (Figure 1B). PCR‐based amplification of the *gyrA* gene produced distinct bands for each strain, confirming strain‐specific growth in the culture (Figure 1C). ProEVs were isolated through ultrafiltration followed by ultracentrifugation, yielding a protein concentration of 4.5 mg/mL. Morphological characterization via TEM showed spherical vesicles (Figure 1D) typical of extracellular vesicles. Dynamic light scattering (DLS) analysis revealed a polydisperse vesicle population with ∼70% centered around 100 nm, spanning a range of 50–150 nm (Figure 1E). 2D and 3D AFM imaging provided further evidence of their dome‐like structure, with vesicle heights of ∼6 nm and lateral diameters of 90–120 nm (Figure 1F–H). Nano particle tracking (NTA) analysis further confirmed that ProEVs possess a nanoscale size distribution, predominantly around ∼80–120 nm, with a slight tail toward larger particles, indicating a largely homogeneous vesicle population with minimal aggregation. The particle concentration was ∼10^1^ ^1^ particles/mL, reflecting efficient isolation. Zeta potential measurements revealed a negative surface charge (approximately −30 to −50 mV), suggesting good colloidal stability and reduced aggregation (Figure S3). After characterization, ProEVs were encapsulated within the polymer matrix (Figure 1I) without altering its thermo‐responsive behavior, as confirmed by no observable shift in LCST post‐encapsulation (Figure 1J). Rheological analysis further validated the thermo‐responsive nature of the formulation. At room temperature (25 °C), the solution exhibited fluid‐like behavior, as evidenced by a higher loss modulus (G″) than storage modulus (G′), ensuring good injectability. Upon heating, a crossover of G′ and G″ occurred at 34 °C, marking the sol‐gel transition (Figure 1K). At temperatures above 34°C, PNHA and PNHA‐ProEVs completely form a gel phase, suggesting the in vivo gel‐forming ability of the polymer formulation (Figure 1L). Loss modulus as a function of shear stress (at constant angular frequency (ω = 1 rad/s, and *T*_gel_ temperature) was studied to confirm the viscous behavior and the strength of the polymer formulations (Figure 1M). A constant region followed by a decline in modulus value helped in determining the threshold limit of linear viscoelasticity (LVE) at T_gel_ for selecting strain for further measurements and determining the effect of injectability on polymer properties. Oscillatory rheology (γ = 1%) at T_gel_ confirmed the viscoelastic stability of PNHA and PNHA‐ProEVs formulations. Both showed a minimum in complex viscosity (∼1 rad/s), followed by an increase at higher ω, reflecting gel‐sol transition and subsequent solid‐like behavior due to water loss and phase separation (Figure 1N).

### 2.2 Stability, Proteomic Characterization, and Functional Assessment of ProEVs

To comprehensively evaluate the performance of PNHA‐ProEVs, we systematically examined their release profile, structural integrity, molecular composition, and biological functionality both in vitro and through proteomic characterization. The release kinetics of ProEVs from the thermo‐gelling PNHA matrix were first evaluated in PBS at 37°C. Upon sol‐gel transition, the formulation exhibited near zero‐order release kinetics over 24 h, achieving a cumulative release of 94 ± 2.45 µg (94 ± 2.45%) of the total loaded vesicles (Figure 2A). Similarly, the encapsulated 5‐ASA also demonstrated sustained release behavior, with approximately 80% cumulative drug release observed within 24 h, supporting the controlled delivery capability of the PNHA matrix (Figure S1D). This release behavior, governed primarily by diffusion followed by gradual matrix erosion, is well suited for localized IBD therapy, ensuring sustained vesicle availability at the inflamed site without systemic exposure. To confirm that vesicles retained their native structure after release, the recovered fraction was visualized by TEM, which demonstrated intact, well‐preserved morphology (Figure 2B). To further verify functional integrity, both fresh (F‐ProEVs) and released ProEVs (R‐ProEVs) were labeled with Calcein‐AM and assessed for their interaction with HT‐29 colonic epithelial cells. Confocal microscopy revealed efficient uptake of both vesicle populations within 3 h, with initial membrane association followed by clear intracellular localization (Figure 2C,D). Biochemical characterization was then performed to assess vesicle composition. Protein and lipid quantification showed comparable levels between fresh (F‐ProEVs) and released ProEVs (R‐ProEVs), indicating that encapsulation and release did not alter their molecular profile (Figure 2E,F). In our strategy, the encapsulation of ProEVs within the PNHA matrix provides a multifaceted platform that enhances reactive oxygen species (ROS) scavenging, as demonstrated by the DPPH assay (Figure 2G). The antioxidant activity of the PNHA‐ProEVs system was further validated through hydrogen peroxide scavenging assays in solvent system (PBS), which showed trends consistent with DPPH (Figure 2H). Further in vitro experiments revealed that treatment of HT‐29 cells with 20 µg of ProEVs significantly enhanced cellular proliferation over 7 days (Figure 2I), while cytotoxicity studies confirmed excellent compatibility of both PNHA and PNHA‐ProEVs formulations with HT‐29 cells (Figure 2J). No morphological alterations were observed, as evidenced by F‐actin and DAPI staining (Figure S1C). Importantly, under TNBS‐induced inflammatory stress, PNHA‐ProEVs markedly improved cell viability (p ≤ 0.0001) compared to untreated controls (Figure 2K), demonstrating their protective effect against inflammation‐driven epithelial damage. Together, these results highlight that PNHA enables efficient encapsulation and sustained release of ProEVs while fully preserving their structural integrity, biochemical composition, and biological activity. The system not only promotes epithelial proliferation, migration, and survival but also offers strong protection under inflammatory conditions, making it highly promising for mucosal repair in IBD. To elucidate the molecular basis underlying these therapeutic effects, we next performed proteomic profiling of ProEVs by mass spectrometry. The MS spectra revealed a diverse protein cargo across the 300–1300 m/z range (Figure 2L). Taxonomic classification identified *Lactobacillus plantarum* as the major contributor, followed by *L. helveticus, L. acidophilus*, and *Streptococcus thermophilus*, all of which are known for their barrier‐supporting, anti‐inflammatory, and immunomodulatory properties (Figure 2M,N). Functional annotation revealed enrichment in proteins associated with protein synthesis (21%), bacterial survival (18%), microbiota modulation (14%), mucosal healing (11%), and immune modulation (8%), alongside additional roles in stress response, DNA repair, and metabolism (Figure 2O and Table S6). Collectively, these results demonstrate that VSL#3‐derived ProEVs released from the PNHA matrix retain structural and functional integrity, exhibit robust bioactivity in vitro, and carry a proteomic repertoire highly relevant to epithelial regeneration, immune modulation, and microbiota stabilization. These findings underscore the therapeutic potential of bioengineered EV‐integrated delivery platforms for localized IBD therapy.

**FIGURE 2:**
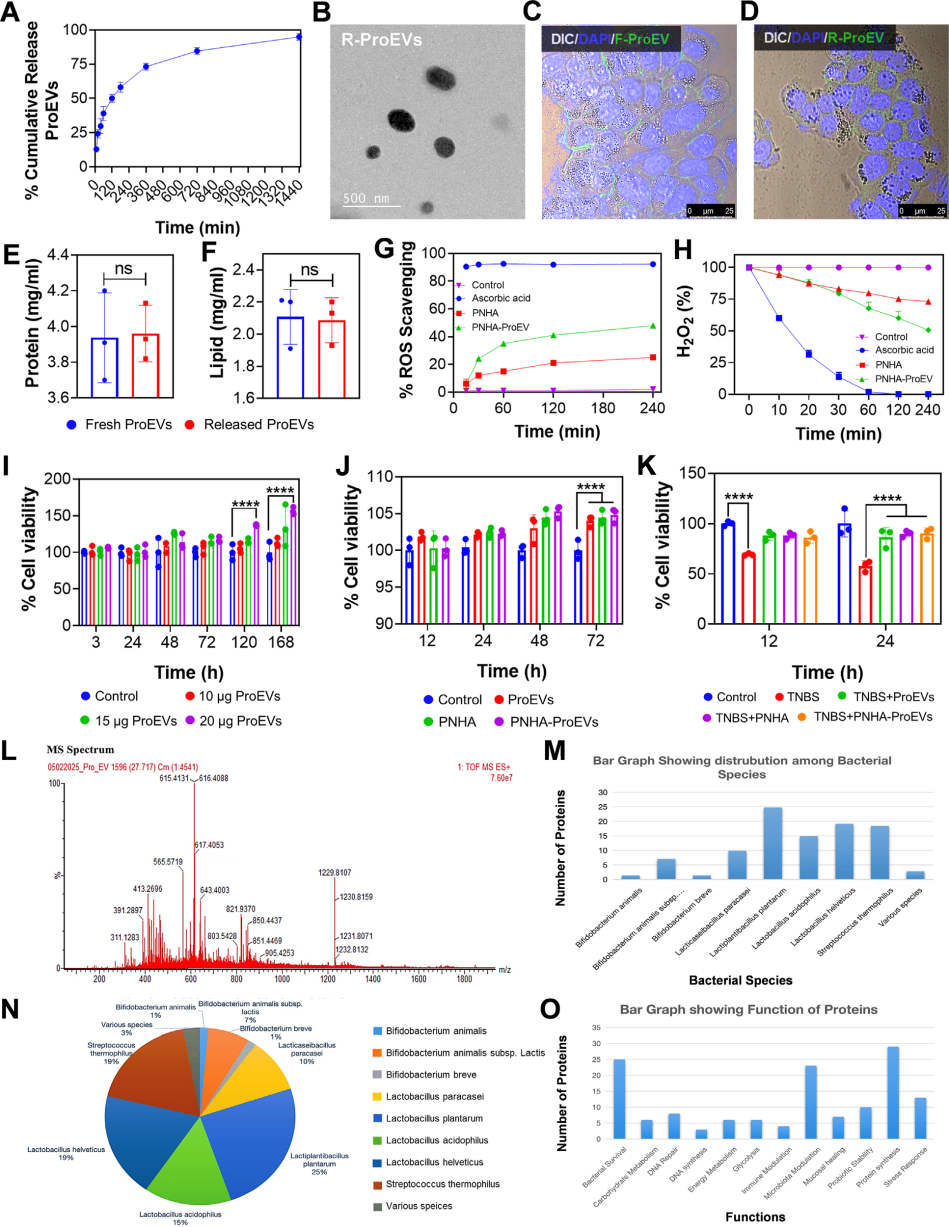
Stability and functional assessment of ProEVs. (A) ProEVs release kinetics from PNHA matrix, (B) Transmission electron micrograph of ProEVs released from PNHA formulation, (C) and (D) Interaction of Calcein‐AM labelled F‐ProEVs (Freshly isolated) and R‐ProEVs (Released from PNHA) with HT 29 cells suggesting integrity of ProEVs post release, (E) and (F) Protein content and lipid content quantification of F‐ProEVs and R‐ProEVs, (G) DPPH assay data plot suggesting antioxidant potential of PNHA‐ProEVs in scavenging ROS, (H) In vitro hydrogen peroxide scavenging assay showing % H_2_O_2_ scavenging by PNHA, ProEVs in different timepoints, (I) Cell viability data plot suggesting cytocompatibility and cell proliferation post treatment with different ProEVs concentrations, (J) Cell viability data plot suggesting optimum cytocompatibility of PNHA and PNHA‐ProEVs, (K) Cell viability data plot suggesting improved cell survival of TNBS‐challenged cells post PNHA‐ProEVs treatment, (L) The mass spectrum (MS) shows a diverse range of peptides/proteins with peaks between m/z 300–1300, indicating a heterogeneous population of proteins in the ProEVs, (M) Bacterial species distribution suggesting ProEVs are enriched in proteins from Lactobacillus plantarum (most dominant), followed by L. helveticus, L. acidophilus, and Streptococcus thermophilus, (N) Pie chart of protein distribution by species showing L. plantarum (25%) and L. helveticus (19%) contribute the most proteins, emphasizing their major role in the bioactivity of ProEVs, (O) Functional categorization of proteins indicating the presence of proteins involved in carbohydrate metabolism, glycolysis, DNA repair, supporting energy and cellular maintenance. Data are presented as mean ± s.d.; n = 3 for all the experiments. ^*^ *p* < 0.05, ^**^ *p* < 0.01, ^***^ *p* < 0.001, ^***^ *p* < 0.0001.

### 2.3 PNHA‐ProEVs Formulation Mitigates Oxidative Stress and Promotes Anti‐Inflammatory Macrophage Polarization

IBD is characterized by significant oxidative stress in the gut mucosa, arising from an imbalance between the production of reactive oxygen species (ROS) and the capacity of endogenous antioxidant systems [31, 32]. This imbalance is exacerbated by immune cell activation, ischemia‐reperfusion injury [33], and gut dysbiosis [34], leading to cellular damage, impaired tissue regeneration [35], and a sustained inflammatory cycle. Addressing oxidative stress is therefore critical in IBD therapy, and antioxidant‐based approaches targeting ROS, inflammation, and microbial dysbiosis hold substantial therapeutic promise. ProEVs are increasingly recognized for their potent antioxidant properties [36], which are attributed to their rich cargo of bioactive molecules including antioxidant enzymes (e.g., superoxide dismutase, catalase), vitamins (C, E), polyphenols, and other regulatory factors [37, 38]. These components synergistically scavenge ROS while also activating intracellular antioxidant defense mechanisms in recipient cells. To harness this potential, we encapsulated ProEVs within a PNHA formulation matrix to enhance their stability, retention, and cellular delivery. The HA backbone also confers targeting capabilities via CD44 receptor interaction [39], improving uptake in inflamed colonic epithelial cells [40], while HA itself contributes additional antioxidant capacity via hydroxyl‐mediated ROS scavenging [41]. The antioxidant efficacy of this formulation was assessed using the HT‐29 cell line exposed to oxidative stress induced by hydrogen peroxide (400 µm for 12 h) (Figure 3A). Post‐exposure treatment with ProEVs, with or without PNHA, significantly improved cell viability, with the combination group showing the highest rescue effect (p = 0.0005), as demonstrated by the viability assay (Figure 3B) and reduced cell death in live‐dead staining images (Figure 3D ***upper panel, 2E)***. Furthermore, a notable decrease in intracellular ROS levels was observed in the PNHA‐ProEVs group as determined by DCF‐DA fluorescence (Figure 3C) (p<0.0001), confirmed visually via fluorescence microscopy (Figure 3D ***, lower panel)***. These findings demonstrate that PNHA‐ProEVs formulation effectively mitigates oxidative stress in intestinal cells, highlighting their therapeutic potential in IBD. Beyond oxidative stress, immune dysregulation in IBD contributes to persistent inflammation [42], largely driven by macrophage‐mediated cytokine release and tissue injury [43]. Therefore, modulating macrophage polarization presents a valuable therapeutic axis. Macrophages exhibit phenotypic plasticity and can transition between pro‐inflammatory (M1) and anti‐inflammatory (M2) states in response to environmental cues [44]. ProEVs are known to influence immune signaling through their microRNA, protein, and lipid cargo, which can promote M2 polarization and suppress M1‐associated markers [45]. Concurrently, HA has also been reported to support M2 polarization via interaction with macrophage surface receptors and its ability to modulate the local inflammatory microenvironment [46, 47]. We hypothesized that the PNHA‐ProEVs formulation would synergistically promote macrophage polarization toward an anti‐inflammatory M2 phenotype (Figure 3F). This was further evaluated using the Griess assay [48] to measure nitric oxide (NO) release from LPS‐stimulated RAW 264.7 macrophages. Treatment with ProEVs alone (p = 0.0003) or in combination with the formulation (p<0.0001) resulted in significant suppression of NO levels compared to the LPS‐only group, indicating reduced M1 activation (Figure 3H). Gene expression profiling further revealed elevated expression of iNOS (Figure 3I) and IL‐6 (Figure 3J) in LPS‐treated cells, which were significantly downregulated upon treatment with ProEVs or the combined formulation. In contrast, IL‐10 (Figure 3K), a key marker of M2 polarization, was significantly upregulated in groups treated with PNHA alone (p = 0.0201) and in the PNHA‐ProEVs (p<0.0001), suggesting a phenotypic shift. These findings were corroborated by immunofluorescence staining for iNOS and CD163, with confocal images confirming increased M2 marker expression and decreased M1 marker expression in treated cells (Figure 3G). Collectively, these results highlight that the PNHA, ProEVs, and PNHA‐ProEVs formulation not only alleviates oxidative stress but also exerts a robust immunomodulatory effect by driving macrophage polarization toward a tissue‐healing M2 phenotype. Flow cytometric analysis of macrophage polarization demonstrated that LPS stimulation markedly increased the proportion of CD86^+^ pro‐inflammatory macrophages, confirming successful induction of the M1 phenotype. In contrast, treatment with ProEVs and PNHA‐ProEVs promoted a shift toward an anti‐inflammatory phenotype, as evidenced by increased CD206 expression and a concomitant reduction in CD86‐positive populations (Figure S4A–F).

**FIGURE 3:**
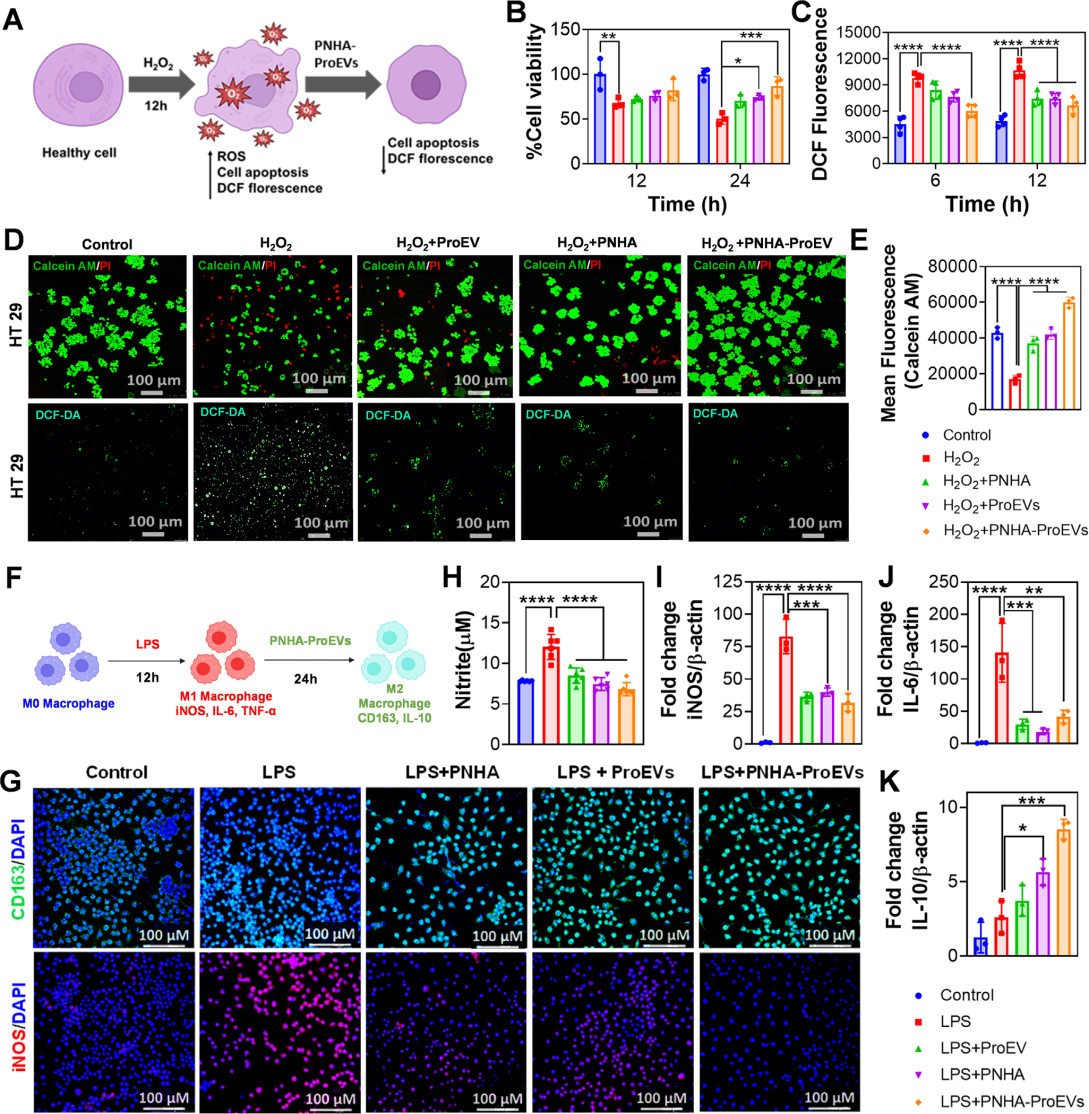
PNHA‐ProEVs formulation enhances epithelial cell survival under oxidative stress and modulates macrophage polarization. (A) Schematic of experimental design outlining antioxidant property evaluation, (B) Viability of HT‐29 cells under oxidative stress post‐treatment with PNHA‐ProEVs, indicating significant cytoprotection, (C) Intracellular ROS levels quantified by DCF‐DA assay showing reduced ROS accumulation upon ProEVs formulation treatment, (D) Calcein‐AM/PI staining (upper row) shows increased live cell population post‐treatment; lower panels show fluorescence images of DCF‐DA‐labeled cells indicating oxidative stress modulation, (E) ImageJ‐based quantification of Calcein‐AM (live cells) staining confirms enhanced survival, (F) Graphical outline of the macrophage polarization, (G) Immunofluorescence staining of iNOS (red) and CD163 (green) in RAW264.7 cells, Elevated iNOS expression was observed in LPS control, whereas treatment groups showed reduced iNOS and enhanced CD163, indicating polarization from M1 (pro‐inflammatory) to M2 (anti‐inflammatory) phenotype. (H) Nitric oxide quantification using Griess assay from RAW264.7 macrophage supernatants shows reduced NO production after ProEVs and PNHA‐ProEVs treatment. (I‐K) RT‐qPCR analysis of iNOS, IL‐6, and IL‐10 expression in LPS‐stimulated RAW264.7 cells indicates suppression of pro‐inflammatory genes (iNOS, IL‐6) and upregulation of anti‐inflammatory IL‐10. Data are presented as mean ± s.d.; n = 3 for all the experiments. ^*^ *p* < 0.05, ^**^ *p* < 0.01, ^***^ *p* < 0.001, ^***^ *p* < 0.0001.

Notably, the comparable in vitro effects of PNHA, ProEVs, and PNHA‐ProEVs likely arise from direct cellular exposure under simplified conditions that do not mimic physiological barriers present in vivo. Therefore, to better evaluate the therapeutic relevance of the combined formulation, we further performed in vivo studies.

### 2.4 PNHA‐ProEVs Formulation Exerts Therapeutic Efficacy in Colitis by Modulating Gut Microbiota and Restoring Intestinal Homeostasis

This study presents a comprehensive evaluation of a bioengineered rectal formulation comprising PNHA‐ProEVs and/or 5‐ASA in a TNBS‐induced colitis rat model (Figure 4A and Table S2). Our findings demonstrate that this combination therapy offers a synergistic and highly effective strategy for mitigating colitis symptoms, restoring epithelial integrity, and modulating the gut microbiota. The therapeutic efficacy was assessed through a comparative analysis of four treatment groups: PNHA alone (Group 3), the formulation loaded with 5‐ASA (PNHA‐5ASA, Group 4), PNHA‐ProEVs formulation (Group 5), and the combination of ProEVs and 5‐ASA within the copolymer (PNHA‐5ASA‐ProEVs, Group 6). Notably, Group 6 exhibited the most significant improvement across all evaluated parameters, suggesting a synergistic effect of the combined formulation (PNHA‐5ASA‐ProEVs). Disease activity index (DAI) scores [49] were substantially reduced across all treatment groups by day 21, indicating resolution of key symptoms such as diarrhea, rectal bleeding, and weight loss (Figure 4B,C). Body weight restoration, a critical clinical indicator of colitis resolution, was markedly improved in Groups 4, 5, and 6, with Group 6 achieving complete recovery by day 21 (p < 0.0001) (Figure 4D). Even the PNHA group (Group 3) demonstrated partial recovery (p  =  0.0121), likely due to the inherent regenerative properties of HA. This highlights HA's contribution to mucosal healing and its utility as a therapeutic scaffold. Moreover, reduction in total leukocyte count suggests a positive response to the therapy (Figure 4E). FITC‐dextran permeability analysis further revealed a marked increase in intestinal leakage in the diseased group, indicating severe barrier disruption. However, treatment with PNHA‐ProEVs significantly reduced FITC‐dextran translocation from gut to serum, demonstrating effective restoration of epithelial integrity (Figure S5). Colon length, another important marker often shortened during colitis due to inflammation and fibrosis, was significantly preserved in the combination therapy group (p < 0.0001, Figure 4F,G). Histological evaluation further supported these findings. Group 6 exhibited reduced mucosal inflammation, lower immune cell infiltration, and restoration of crypt architecture, hallmarks of effective mucosal healing [50] (Figure 4H). These results underscore the complementary roles of ProEVs and 5‐ASA in promoting epithelial regeneration and immune regulation when delivered via the HA‐containing thermo‐responsive formulation. Importantly, the ProEVs used in this study demonstrated a favorable safety profile. Despite originating from Gram‐positive bacteria, they exhibited no hemolytic activity (Figure S1E,F), indicating the absence of inactivity of bacterial hemolysins in the vesicular cargo. This is crucial from a safety standpoint and supports their potential for clinical application. The PNHA formulation serves a dual function: as a protective carrier that enhances ProEVs' bioavailability and as a mucoadhesive, biocompatible scaffold that ensures sustained and localized drug delivery at the site of inflammation. Its ability to scavenge reactive oxygen species (Figure 2G,H), further contributes to reducing oxidative stress and enhancing cell survival at the inflamed mucosal surface. 5‐ASA, a first‐line anti‐inflammatory drug for IBD, plays a pivotal role in the combination therapy by targeting inflammatory cascades in the gastrointestinal tract. Its inclusion in the formulation amplifies therapeutic outcomes through additive and synergistic mechanisms with ProEVs and HA. Together, they create a conducive microenvironment for tissue repair and immune modulation, resulting in better clinical and histological outcomes. Beyond local tissue effects, we also explored the impact of the treatment on the gut microbiota, which is intricately linked to IBD pathogenesis and recovery. Modulation of the microbiome represents a promising approach for achieving long‐term remission in IBD [51]. Using 16S rRNA metagenomic sequencing, we observed pronounced shifts in microbiota composition and diversity in treated animals. The Venn diagram suggests that combination therapy (f) retains most of the beneficial core microbes (112) and adds some unique ones, indicating it may best restore a balanced microbiome. Disease alters the microbiota significantly (b), and PNHA alone (c) has limited restorative effect. The PNHA‐5ASA‐ProEVs treatment group (f) appears most similar to the healthy control (a) in terms of shared taxa and reduced overlap with negative control (b), supporting its therapeutic potential in gut microbiota modulation during colitis (Figure S6A). The highest microbial diversity, an indicator of a healthier and more resilient gut ecosystem [52] was recorded in the group receiving the combined therapy (Group 6), as reflected by increased OTUs (Figure 4I), and diversity indices such as Simpson (Figure S6B) and Shannon (Figure 4J). Taxonomic profiling revealed enrichment of beneficial bacterial families including *Akkermansiaceae*, *Lactobacillaceae*, and certain *Clostridium* clusters (IV and XIVa) (Figure 4K–M). These taxa are known to contribute to gut barrier function, short‐chain fatty acid (SCFA) production, and immune tolerance. *Lactobacillus* species enhance SCFA levels, particularly butyrate, which nourishes colonocytes and exerts anti‐inflammatory effects. *Akkermansia muciniphila* plays a vital role in mucin degradation and SCFA generation, helping to maintain epithelial integrity and reduce bacterial translocation, both of which are often compromised in IBD [53]. *Clostridium* clusters IV and XIVa are associated with regulatory T‐cell induction and immune homeostasis [54]. The observed enrichment of these taxa indicates that PNHA‐ProEVs treatment actively supports microbial reprogramming toward a more anti‐inflammatory and homeostatic state. The stacked bar plot in Figure 4N and Figure S6C–F displays the relative abundance of gut microbiota at phylum, class, order, and genus levels across six experimental groups. The healthy group (a) shows balanced microbiota, while colitis (b) shows dysbiosis. Treatments (c), (d), and (e) offer partial recovery. PNHA‐5ASA‐ProEVs therapy (f) restores beneficial microbes, resembling the healthy state. The heatmap in Figure 4O illustrates genus‐level gut microbiota changes across groups. The healthy control (a) shows high levels of beneficial genera like *Lactobacillus*, *Akkermansia*, and *Muribaculum*, while the colitis group (b) displays dysbiosis with increased *Escherichia*, *Streptococcus*, and *Clostridioides*. Treatments (c), (d), and (e) lead to partial recovery with moderate shifts toward a healthier profile. Notably, the group (f) shows the most restored microbial balance, closely resembling the healthy group with increased beneficial genera and reduced pro‐inflammatory microbes. Compared to traditional probiotics, prebiotics, or fecal microbiota transplantation (FMT), ProEVs offer several critical advantages. Their enhanced stability under harsh gastrointestinal conditions, ability to deliver bioactive cargo directly to mucosal cells, and non‐viable nature reduce the risks associated with live microbial therapies. Furthermore, their immunomodulatory and anti‐inflammatory properties offer a targeted, efficient, and safer alternative for microbiome‐based therapy. In conclusion, our study establishes the better efficacy of a tripartite formulation comprising ProEVs, 5‐ASA, and PNHA for the treatment of colitis. This multimodal approach not only alleviates inflammation and promotes mucosal healing but also reprograms the gut microbiota toward a health‐associated composition. While the inclusion of 5‐ASA enhances the therapeutic effect, the formulation and ProEVs alone hold substantial promise as standalone interventions, offering flexible and patient‐specific treatment options. Future investigations into these components, individually and in combination, may pave the way for personalized, safe, and effective therapies for IBD and related inflammatory disorders.

**FIGURE 4:**
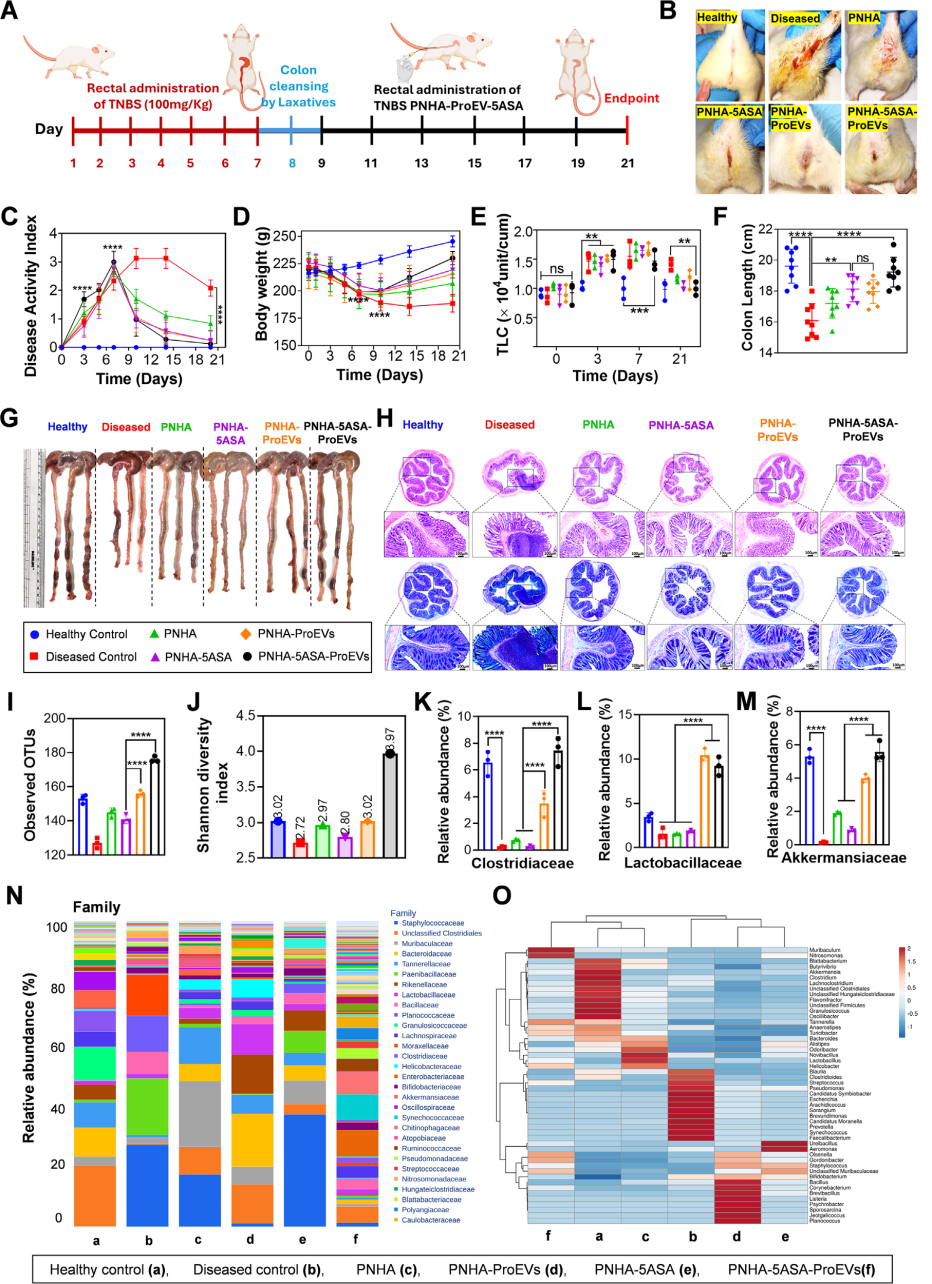
Therapeutic efficacy of PNHA‐ProEVs formulation in TNBS‐induced colitis model. (A) Schematic timeline of the in vivo experimental procedure, (B) Representative digital images of rats depicting rectal bleeding and its attenuation following different treatments, (C) Disease Activity Index (DAI) scores monitored over 21 days post‐induction (n = 8), (D) Daily bodyweight variation across treatment and control groups (n = 8), (E) Total leukocyte count (TLC) measured at multiple timepoints; elevated TLC indicates active inflammation, while reduced TLC post‐treatment reflects therapeutic efficacy (n = 8), (F) Representative images of excised colons from each group showing treatment‐induced recovery (n = 8), (G) Quantitative analysis of colon length, highlighting restoration following PNHA‐ProEVs treatment (n = 8), (H) Representative histological images of colon sections stained with H & E (upper row) and Alcian blue‐PAS (lower row), indicating mucosal restoration and goblet cell preservation (n = 8), (I) Microbiota α‐diversity analysis including: observed OTUs and (J) Shannon index (n = 3), (K‐M) Relative abundance of key bacterial families: Clostridiaceae, Lactobacillaceae, and Akkermansiaceae (n = 3), (N) Family‐level taxonomic distribution presented as percentage of total sequences (n = 3), (O) Heatmap illustrating distinct microbial signatures across treatment groups. Data are expressed as mean ± s.d. Statistical significance determined by one‐way or two‐way ANOVA followed by Tukey's post hoc test. ^*^ *p* < 0.05, ^**^ *p* < 0.01, ^***^ *p* < 0.001, ^****^ *p* < 0.0001.

### 2.5 PNHA‐ProEVs Modulate the Expression of Mucosal Markers and Cytokines

The combination therapy involving ProEVs and/or 5‐ASA along with PNHA has demonstrated a beneficial role in reducing inflammatory markers (Figure 5A). Calprotectin, a calcium‐binding protein found in neutrophils, serves as a biomarker for intestinal inflammation and is elevated in IBD‐like conditions [55]. TNF‐α is a pro‐inflammatory cytokine known to be involved in the pathogenesis of multiple inflammatory diseases, including IBD [56]. We quantified the levels of these to biomarkers in serum samples of the rats at different time points during in vivo study. It was observed that, in groups 5 and 6, the levels of TNF‐α and calprotectin were notably reduced (p <0.0001), akin to basal levels on day 21 (Figure 5B,C). These observations suggest a significant mitigation of inflammation, with the treatment achieving a state similar to the normal baseline levels of these inflammatory markers. Further, we studied the expression levels of some colon‐specific biomarkers and inflammatory mediators in colon tissue harvested from in vivo experiment. The therapy with PNHA‐ProEVs demonstrates a multifaceted role in modulating key markers associated with mucosal integrity and immune regulation. Zonula occludens‐1 (*ZO‐1*, Figure 5J) and Mucin‐2 (*MUC‐2*) are essential components of the intestinal barrier [57], playing crucial roles in maintaining mucosal integrity and preventing epithelial barrier dysfunction. Transforming growth factor‐beta (*TGF‐β*) is a cytokine that plays an important role in immune regulation and tissue regeneration, while interleukin‐10 (*IL‐10*), an anti‐inflammatory cytokine, is known to have anti‐inflammatory and immunomodulatory properties. In group 6 where both 5‐ASA and ProEVs were employed, we observed an approximately 1‐to‐3‐fold increase in the expression of *ZO‐1* (p<0.0001), *IL‐10* (p = 0.0152), *TGF‐β* (p = 0.0054), and *MUC‐2* (p<0.0001), genes in comparison to NC (Figure 5F–I). Moreover, expression levels of some of these genes were also found to be upregulated in group 5 as compared with the negative control, including *MUC‐2* (p = 0.0077), *ZO‐1* (p = 0.0041), and *TGF‐β* (p = 0.0246). Interestingly, in groups 5 and 6, the expression patterns of these genes were rather similar. Furthermore, a significant downregulation of *IL‐6* expression levels was observed among these treatment groups (p<0.0117) (Figure 5D). *TNF‐α* levels were also diminished to some extent in Group 4 (p = 0.0017) and group 5 (p = 0.0001) (Figure 5E). These findings were further corroborated by immunofluorescence imaging of colon tissue, which revealed increased expression of the tight junction protein ZO‐1 (green) and the anti‐inflammatory macrophage marker CD163 (green), along with reduced expression of the pro‐inflammatory marker iNOS (red) in treatment groups 5 and 6 (Figure 5K). These findings indicate suppression of inflammatory cascades and restoration of mucosal integrity. Quantitative analysis of the images by ImageJ software further supports this anti‐inflammatory and regenerative effect (Figure 5L–N). These findings support that ProEVs possess anti‐inflammatory properties, modulating immune responses and reducing inflammatory cytokine levels. Additionally, PNHA, through its controlled drug release mechanism and tissue regeneration properties, further contributes to the reduction of inflammation. The combination of PNHA, ProEVs, and 5‐ASA synergistically targets different aspects of the inflammatory cascade, resulting in a more pronounced reduction in serum calprotectin and TNF‐α levels compared to individual treatments. This reduction likely reflects a comprehensive modulation of the mucosal cytokine milieu and dampening of the inflammatory response, leading to improved disease outcomes and better overall health in individuals with inflammatory conditions. This comprehensive modulation of mucosal markers and cytokine expression underscores the therapeutic potential of the combination therapy in treating inflammatory conditions, particularly those affecting the gastrointestinal tract, and highlights its role in promoting mucosal healing and immune regulation.

**FIGURE 5:**
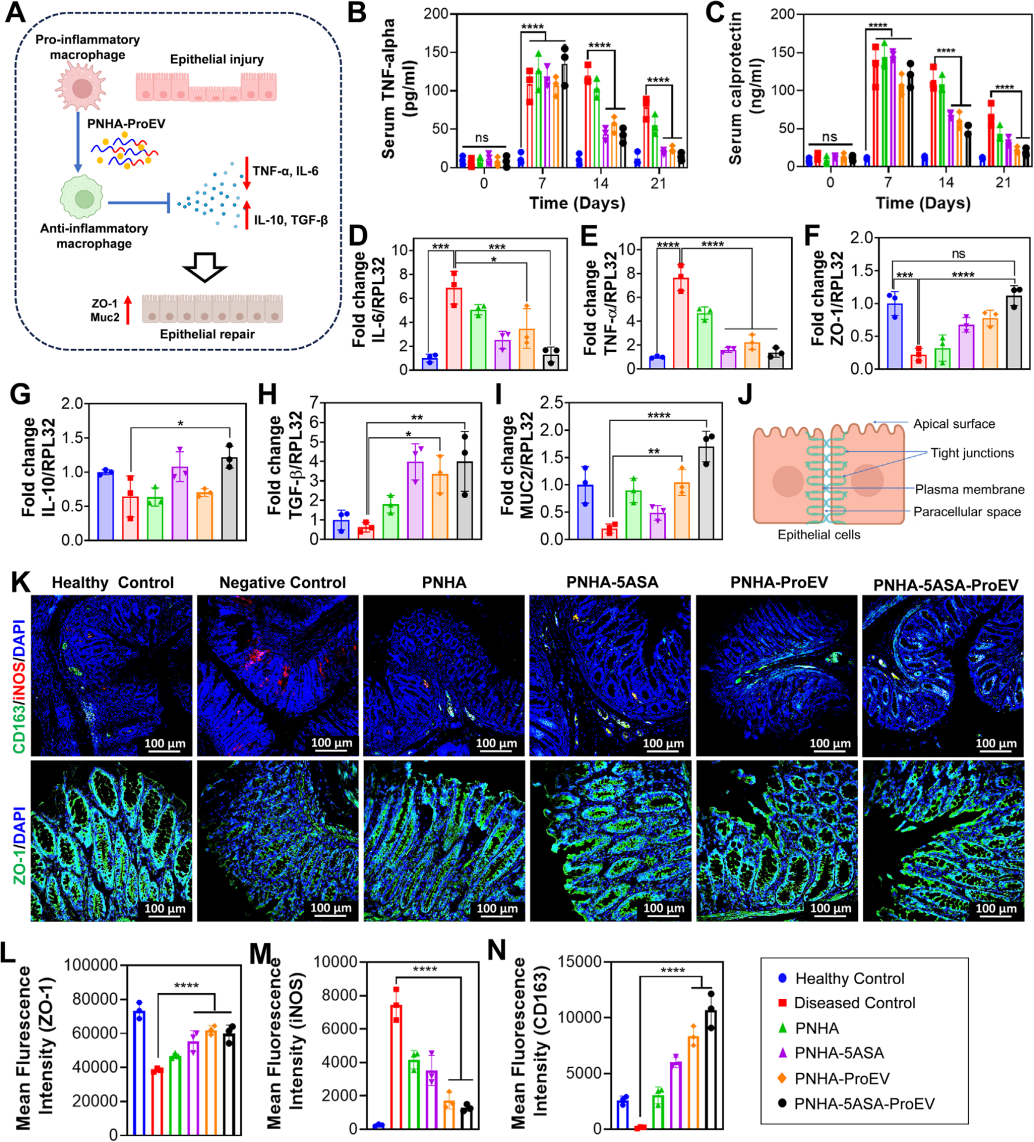
PNHA‐ProEVs modulates the expression of cytokines and mucosal biomarkers. (A) Graphical illustration displaying the immune modulation and gut barrier repair by ProEVs (B) Rat serum samples were analyzed for the TNF‐α, (C) calprotectin levels at different timepoints, (D) Post sacrifice, colon tissues were excised and analyzed for the mRNA levels some colon specific biomarkers and inflammatory mediators IL‐6, (E) TNF‐α, (F) ZO‐1, (G) IL‐10, (H) TGF‐β, (I) MUC‐2, (J) representative image of tight junctions in intestinal epithelium, (K) The presence of M1 and M2 macrophages in colon sections was visualized by immunofluorescence stating of iNOS (Red) and CD163 (Green) using confocal microscopy, also the intestinal barrier repair was assessed by visualizing the expression pattern of tight junction protein ZO‐1 (Green), (L–N) Quantification of fluorescence intensity of ZO‐1, iNOS and CD163 by ImageJ software. Shown are the representative images from 5 slides with n = 5 biologically independent animals. Data are presented as mean ± s.d. ^*^ *p* < 0.05, ^**^ *p* < 0.01, ^***^ *p* < 0.001, and ^****^ *p*<0.0001.

### 2.6 Enhanced Efficacy of PNHA‐ProEVs Rectal Formulation Over Oral and Rectal Probiotics in Restoring Gut Barrier Integrity and Microbiota

To rigorously assess the therapeutic potential of the PNHA‐ProEVs formulation, we conducted a comparative study against both oral and rectal administration of the VSL#3 probiotic in a TNBS‐induced colitis rat model (Figure 6A and Table S5). Over a 21‐day treatment period, rats receiving the PNHA‐ProEVs formulation exhibited significantly (p < 0.0001) improved clinical outcomes, as evidenced by reduced body weight loss and DAI scores when compared to both oral and rectal VSL#3‐treated groups (Figure 6B,C). While rectal VSL#3 formulated in PNHA showed moderate improvement over oral delivery, it remained significantly less effective than the PNHA‐ProEVs system in mitigating colitis symptoms. Colon length, a recognized marker of inflammation and fibrosis in colitis models, was significantly preserved in the PNHA‐ProEVs group. In contrast, oral VSL#3 administration resulted in pronounced colon shortening, while rectal VSL#3 achieved moderate preservation (Figure 6D,E). Histopathological evaluation via H&E staining revealed minimal mucosal ulceration, reduced inflammatory cell infiltration, and restored epithelial structure in the PNHA‐ProEVs‐treated group (Figure 6F, *upper panel)*. Furthermore, Alcian blue‐PAS staining demonstrated substantial preservation of goblet cells and mucin‐secreting epithelium hallmarks of mucosal healing predominantly in the PNHA‐ProEVs group, with only partial restoration observed in the rectal PNHA‐VSL#3 group and negligible effects in the oral VSL#3 group (Figure 6F, *lower panel)*. The enhanced therapeutic efficacy of the PNHA‐ProEVs system is attributed to the synergistic benefits of the HA‐based thermo‐responsive formulation and the bioactive ProEVs payload. The formulation facilitates local retention, controlled release, and targeted delivery to inflamed CD44‐overexpressing tissues. Simultaneously, ProEVs contribute antioxidant and immunomodulatory molecules that attenuate oxidative stress and modulate immune responses at the site of inflammation. In contrast, oral VSL#3 likely undergoes degradation in the upper GI tract, leading to inefficient delivery and reduced therapeutic outcomes. 16S rRNA metagenomic analysis further validated the microbiome‐modulating potential of ProEVs. The Venn diagram illustrated greater overlap in microbial species between the ProEVs and healthy groups, suggesting more effective microbiome restoration compared to oral VSL#3 (Figure 6G). This indicates that rectal delivery, particularly of ProEVs, not only reinstates a broader microbial community but also supports a more functionally resilient gut ecosystem. Taxonomic profiling revealed that ProEVs and rectal VSL#3 promoted the growth of beneficial bacteria such as *Bifidobacterium* and *Lactobacillus*, whereas oral VSL#3 failed to enhance their abundance (Figure 6H and Figure S7). The heatmap illustrates, diseased group (B) exhibits a distinct microbial signature with elevated levels of pathogenic or inflammation‐associated genera like *Butyricicoccus*, *Faecalibaculum*, and *Prevotella*, and a reduction in beneficial genera such as *Lactobacillus*, *Faecalibacterium*, and *Roseburia*. Treatment groups (C, D, E) show partial restoration of microbial balance, with rectal ProEVs (E) showing the most similar profile to the healthy group (A), indicating enhanced normalization of gut microbiota, further confirming the improved microbiome‐restoring effect of PNHA‐ProEVs compared to oral or rectal VSL#3 (Figure 6I). Both PNHA‐ProEVs and PNHA‐ VSL#3 treatments resulted in significantly higher OTUs (Figure 6N) and α‐diversity compared to oral VSL#3, as shown by Chao, Fisher, Simpson, and Shannon diversity indices (Figure 6J–M). These findings indicate a more balanced and diverse microbial community, critical for gut homeostasis and immune regulation. The abundance of specific bacterial genera was also assessed, particularly the VSL#3‐associated genera *Streptococcus, Bifidobacterium*, and *Lactobacillus* (Figure 6O–R). Notably, the PNHA‐ProEVs and PNHA‐VSL#3 groups showed a significant increase in the relative abundance of these genera, with PNHA‐ProEVs yielding the most pronounced effect. KEGG‐based functional profiling revealed severe metabolic impairment in the Diseased group compared to Healthy controls. Oral or PNHA‐VSL#3 treatments partially restored microbial functions, while PNHA‐ProEVs achieved near‐complete recovery, with enrichment of metabolic, genetic, and immune‐regulatory pathways. These results highlight the enhanced ability of rectally delivered ProEVs to re‐establish microbial homeostasis and functional resilience over conventional probiotic approaches (Figure S8). Together, these findings demonstrate that the PNHA‐ProEVs formulation provides improved therapeutic benefits compared to both oral and PNHA‐VSL#3. By enabling localized, sustained delivery of immunomodulatory and antioxidant‐rich ProEVs, this platform effectively reduces inflammation, promotes mucosal healing, and restores microbial diversity and functionality. This bioengineered system offers a promising strategy for targeted and responsive therapy in IBD.

**FIGURE 6:**
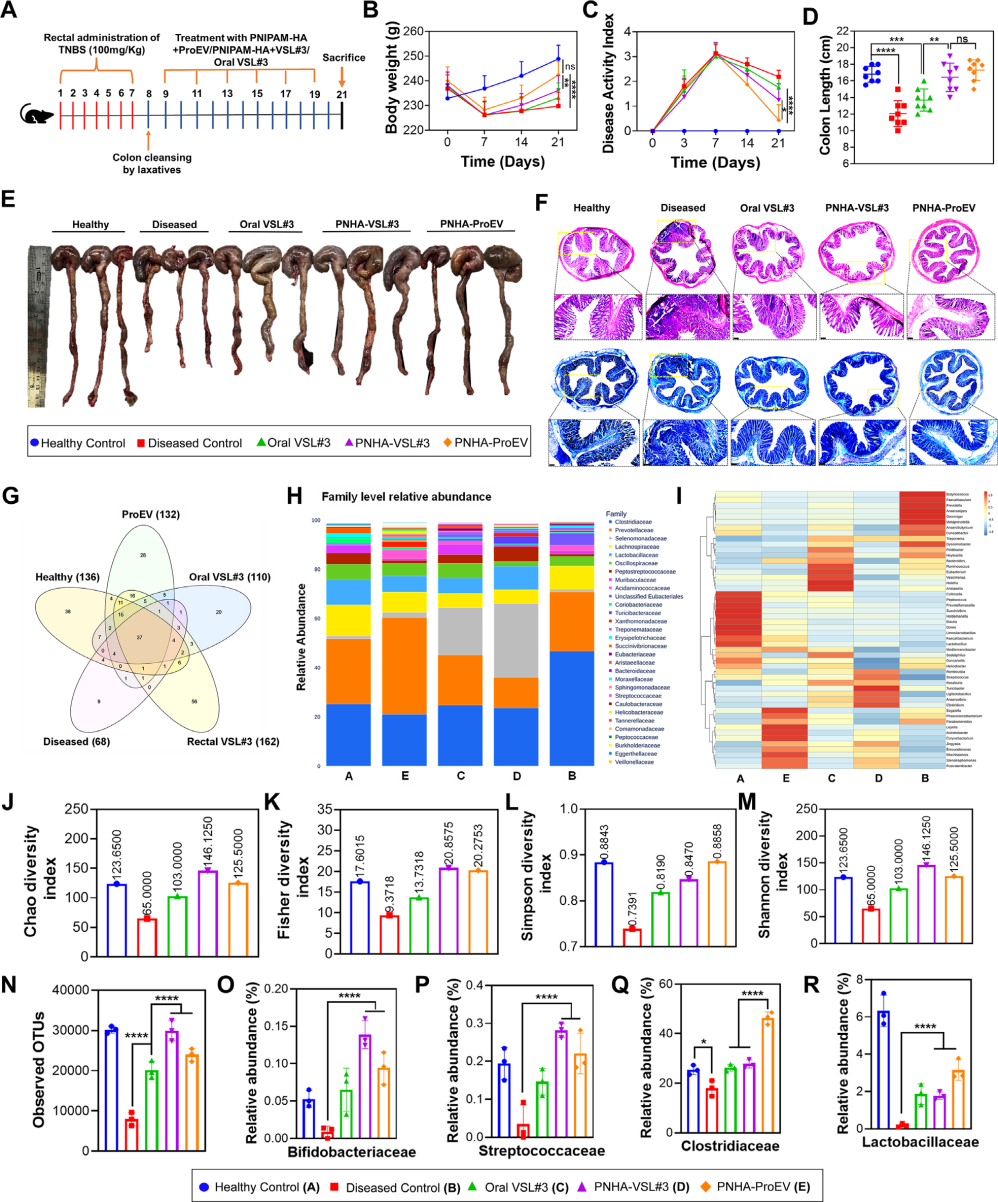
PNHA‐ProEVs show enhanced therapeutic efficacy in colitis over oral and rectal VSL#3. (A) Experimental timeline for in vivo experiment, (B) Daily bodyweight changes in each group for 21 days, (C) Disease Activity Index (DAI) score data plot for 21 days, (D) and (E) Digital images and data plot showing changes in colon lengths post sacrifice, (F) Histology images of rat colon sections stained with hematoxylin and eosin (Upper row) and alcian blue‐PAS (Bottom row). (G) Rat fecal samples were collected on Day 21 and analyzed for gut microbiome by 16S rRNA sequencing, Venn diagram representing the number of overlapping and unique bacterial species across different experimental groups, (H) Family‐level taxonomy is presented as a percentage of total sequences, (I) Heatmap demonstrating distinct microbial signatures across treatment groups, (J) Chao diversity index, (K) Fisher diversity index (L) Simpson diversity index and (M) Shannon diversity index, representing the α diversity, (N) Estimation of microbial community observed OTU (operational taxonomic unit) richness, (O)‐(R) % Relative abundance of beneficial families Bifidobacteriaceae, Streptococcaceae, Clostridiaceae, and Lactobacillaceae. Data are presented as mean ± s.d. from a representative experiment, ^*^ *p* < 0.05, ^**^ *p* < 0.01, ^***^ *p* < 0.001, ^****^ *p* < 0.0001, analyzed by one‐way or two‐way ANOVA with Tukey's HSD multiple comparison post hoc test.

### 2.7 PNHA‐ProEVs Formulation Outperforms Oral and Rectal VSL#3 in Reducing Inflammation and Restoring Epithelial Barrier

Restoring intestinal immune balance and repairing the epithelial barrier are critical therapeutic goals in managing IBD. In this study, we compared the therapeutic efficacy of PNHA‐ProEVs against standard oral and PNHA‐VSL#3 treatments in a murine model of colitis. As illustrated in Figure 7A, PNHA‐ProEVs therapy synergistically attenuated inflammation and supported gut barrier regeneration via immune modulation and mucosal repair. Serum TNF‐α levels (Figure 7B) and fecal calprotectin (Figure 7C), two key clinical markers of active intestinal inflammation, were significantly reduced in animals treated with PNHA‐ProEVs compared to those receiving oral or PNHA‐VSL#3. Notably, these reductions were evident across multiple time points, indicating sustained anti‐inflammatory effects. To assess epithelial integrity and immune homeostasis, post‐mortem colon tissues were evaluated for the expression of tight junction and mucin‐associated markers. Quantitative RT‐PCR analysis revealed significant upregulation of epithelial markers *ZO‐1* (Figure 7D) and *MUC2* (Figure 7E), alongside a favorable immunomodulatory cytokine profile marked by suppressed *IL‐6* (Figure 7F) and enhanced *IL‐10* (Figure 7G) expression in the PNHA‐ProEVs group. These results point toward restoration of epithelial barrier function and mitigation of the pro‐inflammatory milieu. Furthermore, immunofluorescence staining confirmed increased expression of tight junction protein ZO‐1 (green) and reduced VEGF (red) levels in the PNHA‐ProEVs‐treated group compared to the high VEGF observed in the negative control, suggesting both epithelial restoration and resolution of inflammation‐induced angiogenic remodeling commonly seen in chronic colitis. Additionally, polarization of macrophages toward an anti‐inflammatory M2 phenotype was observed, as indicated by elevated CD163 (green) and reduced iNOS (red) expression (Figure 7H), supporting the role of ProEVs in orchestrating a favorable local immune environment. The immunofluorescence images were further quantified using ImageJ software to validate these observations quantitatively (Figure 7I–L). Together, these findings underscore the improved therapeutic potential of ProEVs delivered via the thermo‐responsive PNHA platform over conventional probiotic treatments. This integrated approach offers a promising direction for localized, cell‐free IBD therapy that targets both inflammation and mucosal regeneration.

**FIGURE 7:**
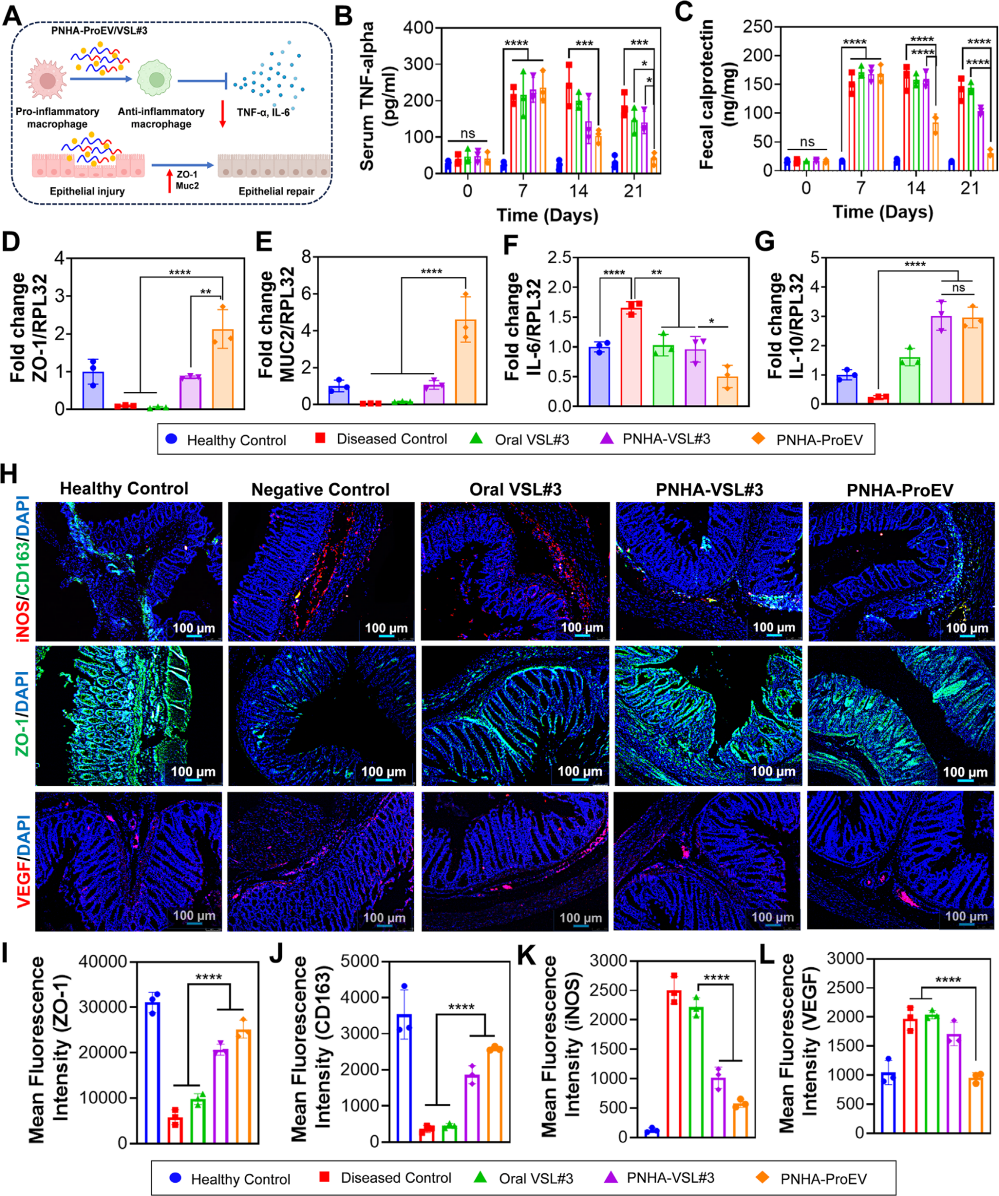
PNHA‐ProEVs outperform oral and rectal VSL#3 in reducing inflammation and restoring the epithelial barrier. (A) Graphical illustration displaying the immune modulation and gut barrier repair by ProEVs, (B) serum TNF‐α and (C) fecal calprotectin were quantified levels at different timepoints, (D) Post sacrifice, colon tissues were excised and analyzed for the mRNA levels some colon specific biomarkers and inflammatory mediators *ZO‐1*, (E) *MUC 2*, (F) *IL‐6*, (G) *IL‐10*, (H) The presence of M1 and M2 macrophages in colon sections was visualized by immunofluorescence stating of iNOS (Red) and CD163 (Green) using confocal microscopy, also the intestinal barrier repair and regeneration was assessed by visualizing the expression pattern of tight junction protein ZO‐1 (Green) and vascular endothelial growth factor (VEGF) (Red), (I)‐(L) ImageJ quantification of ZO‐1, iNOS, CD163 and VEGF. Data are presented as mean ± s.d. ^*^ *p* < 0.05, ^**^ *p* < 0.01, ^***^ *p* < 0.001, and ^****^ *p*<0.0001.

### 2.8 Transcriptomic Insights Reveal Immunomodulatory and Regenerative Mechanisms Underlying ProEVs Efficacy

To elucidate the molecular mechanisms underlying the therapeutic efficacy of PNHA‐ProEVs, we performed comprehensive transcriptomic profiling and differential gene expression analysis across key comparisons: Healthy *vs* Diseased (Figure S9A,B), Diseased *vs* PNHA‐ProEVs (Figure S9C,D), and Healthy *vs* PNHA‐ProEVs colonic tissues (Figure S9E,F). In the Healthy *vs* Diseased comparison (Figure 8A–C), Diseased samples cluster distinctly from healthy controls, reflecting extensive transcriptomic reprogramming during colitis. Diseased tissue shows elevated expression of immune and inflammation‐related genes (e.g., *Ccl5, Gbp4, Fcrls, Zbp1*), along with stress‐associated epithelial markers (*Aqp8, Fabp1, Lgals3*), indicating immune activation, epithelial stress, and barrier disruption [58, 59]. In contrast, healthy tissues display higher expression of genes involved in epithelial differentiation, metabolism, and barrier maintenance (e.g., *Slc13a1, Aldh1a1, Mink1, Plxnb3*) [60]. This shift from homeostatic to inflammatory gene programs underscores cytokine‐driven immune‐epithelial crosstalk in colitis and establishes a molecular baseline for evaluating therapeutic restoration. (Figure 8A). Functional enrichment of downregulated genes shows marked suppression of inflammation, hypoxia, and stress‐related pathways following treatment. Reduced immune signaling, cytokine activity, and cytoskeletal remodeling, together with inhibition of *TNF, IL‐17, NF‐κB, MAPK*, and *HIF‐1* pathways, indicate attenuation of immune activation and restoration toward intestinal homeostasis [61] (Figure 8B). Functional enrichment of upregulated genes highlights activation of regenerative and immune‐resolving pathways that support restoration of intestinal homeostasis. Enhanced epithelial remodeling, barrier repair, and metabolic adaptation, along with regulated activation of *PI3K‐Akt, MAPK, HIF‐1*, and cytokine signaling pathways, indicate a shift from inflammatory injury toward mucosal healing and immune normalization following treatment [62] (Figure 8C). Strikingly, treatment with the PNHA‐ProEVs formulation induced a substantial reprogramming of the colonic transcriptome (Figure 8D–F). PNHA‐ProEVs treatment induced a coordinated transcriptomic shift characterized by suppression of immune activation and chemokine signaling (e.g., *Zbp1, Ido1, Ccr2b, Cxcr2b*) alongside robust upregulation of epithelial repair and antimicrobial genes (*Reg3b, Reg3g, Regb1*). This integrated immune‐epithelial reprogramming supports inflammatory resolution, restoration of barrier integrity, and mucosal healing in colitis. (Figure 8D). GO and KEGG analyses show significant downregulation of genes involved in TNF‐α, LPS, bacterial, and hypoxia responses, indicating attenuation of cytokine‐driven inflammation and innate immune activation. Reduced pathways related to leukocyte migration, chemokine signaling, antigen presentation, and Th1/Th17 responses reflect diminished immune cell recruitment and immune resolution, creating a permissive environment for mucosal healing and restoration of intestinal homeostasis (Figure 8E). Functional enrichment of upregulated genes in the Diseased *vs* PNHA‐ProEVs comparison reveals a shift toward epithelial repair, metabolic reprogramming, and cellular restoration following treatment. Enrichment of metabolic, transcriptional, and mitochondrial pathways, together with activation of oxidative phosphorylation and biosynthetic processes, indicates recovery of epithelial function and energetics, supporting active tissue regeneration and durable mucosal healing (Figure 8F). Comparison between Healthy and PNHA‐ProEVs‐treated tissues revealed minimal transcriptomic disparity, underscoring the restorative potential of the PNHA‐ProEVs formulation (Figure 8G–I). Hierarchical clustering of DEGs shows that PNHA‐ProEVs treatment induces a distinct reparative transcriptional program, separate from both healthy and diseased states, indicating active therapeutic reprogramming. Moderated stress and immune signaling, together with upregulation of epithelial maintenance, metabolic, and transcriptional regulators (e.g., *Echs1, Hdac3, Hic1*), support a pro‐homeostatic state that balances immune resolution with epithelial regeneration (Figure 8G). Functional enrichment of downregulated genes in PNHA‐ProEVs‐treated *vs* healthy tissue indicates suppression of transport, metabolic, and stress‐associated programs, reflecting a regulated, low‐activation mucosal state. Reduced membrane transport, oxidative processes, and detoxification pathways suggest stabilization of epithelial physiology and normalization of metabolic demand following therapy (Figure 8H), Functional enrichment of upregulated genes in PNHA‐ProEVs *vs* healthy tissue indicates controlled activation of protective and regenerative programs without inflammatory signatures. Enhanced mitochondrial organization, oxidative phosphorylation, and stress‐adaptive pathways suggest improved epithelial bioenergetics and resilience supporting barrier maintenance and tissue repair (Figure 8I). Taken together, these transcriptomic findings provide strong molecular evidence that ProEVs delivered via a mucoadhesive, thermo‐responsive formulation system not only suppress pro‐inflammatory signaling but also promote angiogenic repair, epithelial barrier restoration, and immune reprogramming. This dual‐action therapeutic effect, anti‐inflammatory and regenerative, confers a significant advantage over conventional probiotic or mono‐therapeutic approaches, highlighting the translational promise of this bioengineered extracellular vesicle platform for IBD management.

**FIGURE 8:**
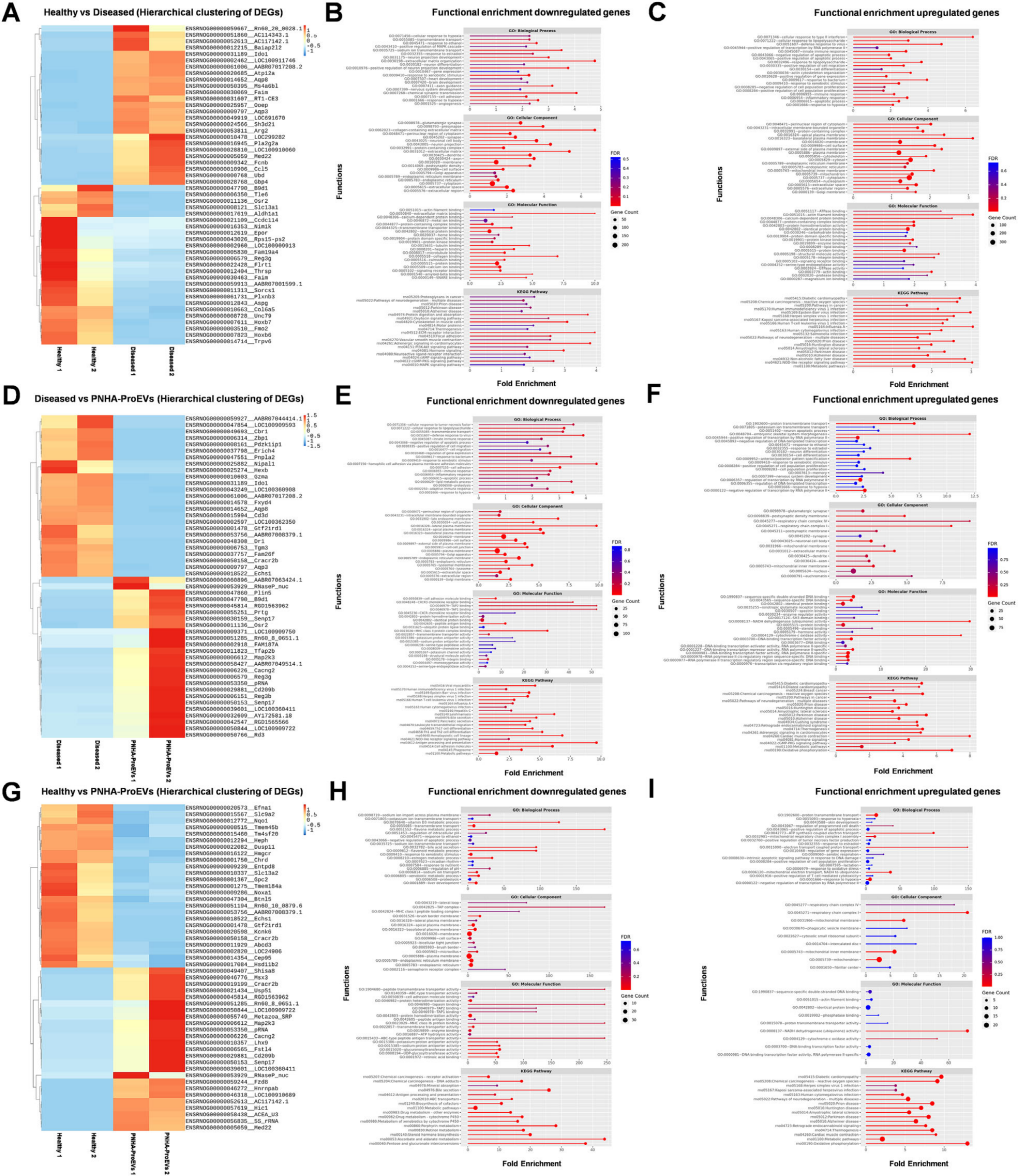
ProEVs‐induced transcriptomic changes support vascular regeneration, epithelial barrier restoration, and immunological homeostasis. (A) Heatmap of DEGs shows strong clustering of inflammation‐related genes in the diseased group (e.g., *Il1b, Nos2, Ccl2, TNF‐α*) are highly upregulated. (B) Downregulated pathways of healthy control show enrichment in metabolic and epithelial barrier functions that are lost in colitis. Notable downregulation in tight junction, oxidative phosphorylation, and fatty acid metabolism, (C) Upregulated pathways of diseased group has high expression of genes involved in cytokine‐cytokine receptor interaction, NF‐κB signaling, TNF signaling, and chemokine signaling, indicating heightened inflammation and immune activation, (D) Heatmap of DEGs demonstrating PNHA‐ProEVs downregulates many of the same inflammatory markers that were elevated in disease (e.g., *TNF, Nos2, Il6, Cxcl1*), indicating transcriptional reversal, (E) PNHA‐ProEVs treatment significantly downregulates inflammatory pathways, including *TNF, IL‐17*, and NOD‐like receptor signaling, (F) PNHA‐ProEVs treatment upregulates expression of genes involved in tight junction formation, oxidative phosphorylation, peroxisome function, and fatty acid metabolism, suggesting improved epithelial barrier integrity and resolution of inflammation, (G) Heatmap of DEGs suggest PNHA‐ProEVs treated group closely clusters with healthy controls. Only a few residual gene expression differences remain, highlighting therapeutic restoration. (H) & (I) Functional profiles of PNHA‐ProEVs‐treated tissues are nearly identical to healthy, with low inflammatory gene activity and strong enrichment in barrier repair, lipid metabolism, and immune regulation.

### 2.9 Long‐Term In Vivo Biocompatibility Evaluation of PNHA

The long‐term in vivo biocompatibility of biomaterials is a critical prerequisite for their successful translation into clinical applications, as systemic or local adverse responses can significantly limit their therapeutic utility. In this study, the PNHA formulation was implanted into dorsal muscle pouch of Wistar rats to evaluate its safety profile in comparison to PBS controls (Figure 9A). Throughout the two‐week observation period, no behavioral abnormalities, blood profile (Figure 9B), weight loss (Figure 9L), or signs of distress were recorded, indicating that the formulation did not elicit any overt systemic toxicity. Biochemical analyses of serum markers including urea, albumin, bilirubin, creatinine, SGOT, SGPT, and triglycerides, remained within the normal physiological range in PNHA‐treated animals, comparable to PBS controls (Figure 9C–I, *No Significant Difference)*. These findings suggest that PNHA did not interfere with renal or hepatic function, both of which are primary clearance pathways for exogenous materials. Importantly, the absence of alterations in metabolic indicators reinforces the systemic safety of the formulation. Histopathological examination further supported the biocompatibility of PNHA. H&E‐stained sections of major organs (liver, lungs, kidneys, heart, spleen, thymus, and colon), as well as the local muscle implantation site, revealed preserved tissue architecture without evidence of necrosis, inflammatory infiltrates, or fibrosis (Figure 9J,K). No organomegaly was observed, and organ weights remained comparable to those of the PBS control group, further excluding the possibility of chronic inflammatory or degenerative responses (Figure 9M–P, *No Significant Difference)*. Similarly, repeated rectal administration of PNHA over 15 days did not induce any significant changes in the above‐mentioned biochemical parameters or colonic histology, indicating excellent local and systemic tolerability (Figure S10). The lack of local inflammatory reaction at the implantation site indicates favorable host‐material interactions, an essential feature for materials intended for therapeutic use in sensitive environments such as the intestinal mucosa. These results align with previous reports demonstrating the biocompatibility of PNIPAM‐based and HA‐modified formulations, where minimal immune activation and absence of systemic toxicity have been noted [63, 64]. The incorporation of hyaluronic acid in PNHA likely contributes to its favorable tissue compatibility, as HA is an endogenous polysaccharide involved in wound healing and immune regulation [65]. Collectively, our findings demonstrate that PNHA possesses an excellent safety profile over the tested duration, supporting its suitability for further preclinical evaluation in long‐term therapeutic applications.

**FIGURE 9:**
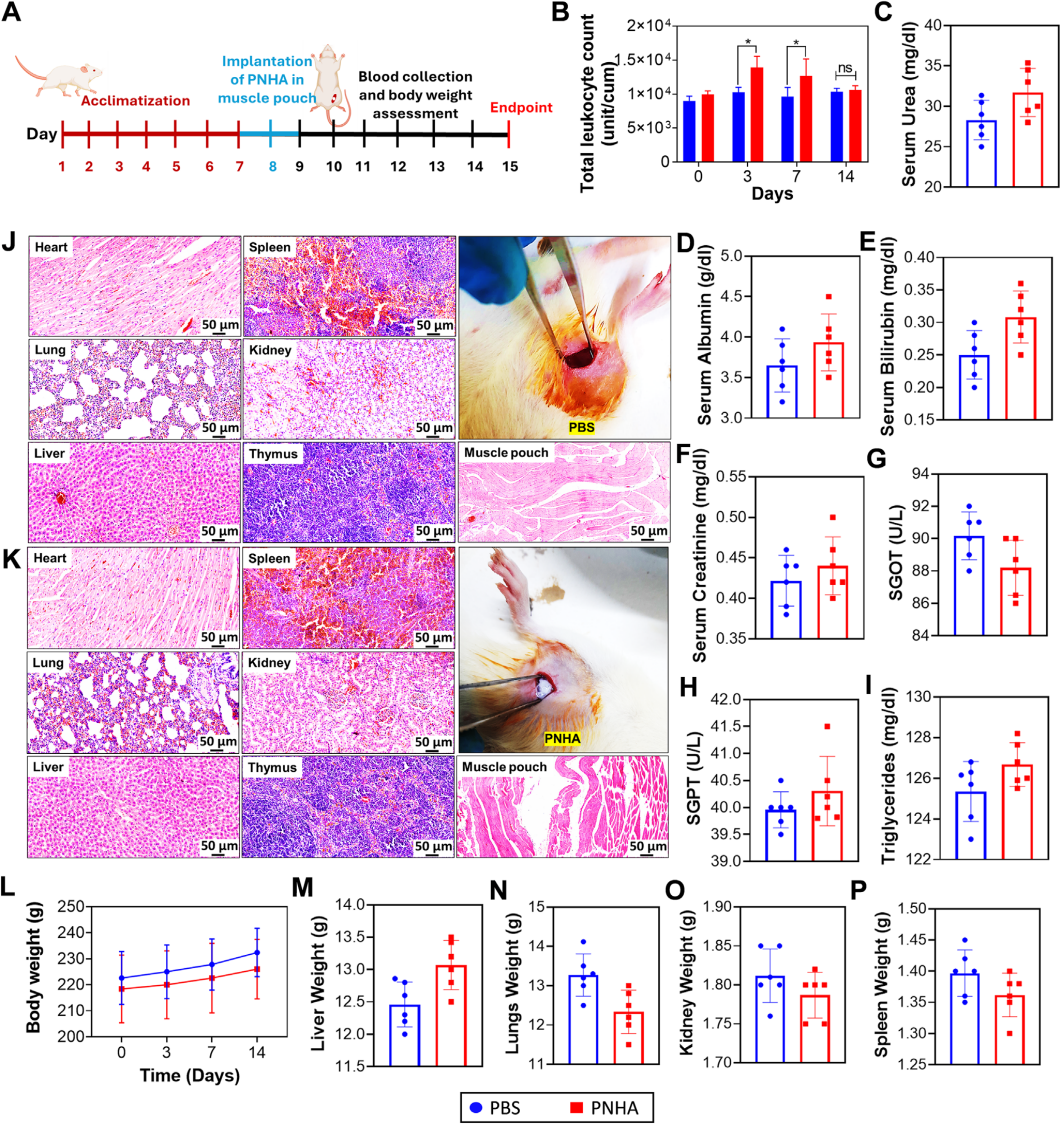
Long‐term in vivo biocompatibility assessment of PNHA. (A) In vivo toxicity assessment work plan (B) Data plot showing total leukocyte count of rats on different days, (C–I) biochemical parameters urea, albumin, bilirubin, creatinine, SGOT, SGPT, and triglycerides quantified in rat blood serum, (J) & (K) Histological analysis of major organs including heart, spleen, lung, kidney, live thymus ang muscle pouch on day 14 of PNHA implantation, (L) Data plot for body weight of rats on different days suggesting no negative impact of PNHA on body weight, (M)‐(P) weight of vital organs liver, lungs, kidney and spleen post sacrifice suggesting no organomegaly post PNHA implantation.

### 2.10 Off‐the‐Shelf Therapeutic Potential of PNHA‐ProEVs Ensuring Clinical Readiness

A critical step in the clinical translation of extracellular vesicle (EV)‐based therapeutics is ensuring their stability during storage and distribution, without compromising their biological activity. Unlike freshly prepared vesicle formulations, which may suffer from rapid degradation, loss of membrane integrity, or reduced bioactivity upon extended storage, lyophilization offers a viable strategy to transform lab‐scale preparations into off‐the‐shelf products with longer shelf life and easier handling [66, 67]. In the present study, we investigated the stability profile of ProEVs incorporated within our thermo‐responsive polymer formulation under two conditions: (i) immediately after lyophilization and reconstitution, and (ii) following six months of storage at 4°C (Figure 10A,B). The lyophilized (LF) and stored formulations (LSF) displayed comparable physical characteristics to the freshly prepared PNHA‐ProEVs (FF). LCST determination revealed no significant difference among fresh, lyophilized, and stored samples, confirming the thermo‐responsive behavior of the copolymer was preserved (Figure 10C). Release studies demonstrated that ProEVs were steadily liberated from all three formulations, suggesting that the polymer matrix remained functionally competent in controlling vesicle delivery (Figure 10D). Further characterization of the released ProEVs by SEM (Figure 10E,F), DLS (Figure 10G,H) and zeta potential showed that ProEVs from lyophilized and stored matrices exhibited slightly reduced diameters (∼50–60 nm) compared to fresh vesicles, which may be attributed to vesicle shrinkage during lyophilization. However, the addition of trehalose likely minimized structural collapse, as vesicles remained intact with stable zeta potential values (−18 mV). Importantly, protein and lipid quantification demonstrated no significant differences across the three groups (FF, LF and LSF), indicating that lyophilization and storage did not compromise the molecular composition of ProEVs (Figure 10I,J). The bio‐functional activity of the formulations was also retained post‐lyophilization. DPPH assays revealed comparable antioxidant activity (45%–50%) among FF, LF, and LSF (Figure 10K). In HT‐29 cells, treatment with all three formulations led to reduced intracellular ROS, as reflected by decreased DCF fluorescence in H_2_O_2_‐challenged cells, while simultaneously promoting cell survival (Figure 10L,M). These findings were further corroborated by fluorescence imaging with DCFDA and CAM/PI staining, which confirmed reduced oxidative stress and preserved cell viability in treated groups (Figure 10N). Collectively, these results demonstrate that lyophilization, when supported with trehalose as a cryoprotectant, preserves the physical, biochemical, and bio‐functional properties of PNHA‐ProEVs even after six months of storage at 4°C. The ability to maintain vesicle integrity, thermo‐responsive polymer behavior, and antioxidant/epithelial protective functions underscores the potential of this formulation as an off‐the‐shelf therapeutic platform for clinical translation in IBD.

**FIGURE 10:**
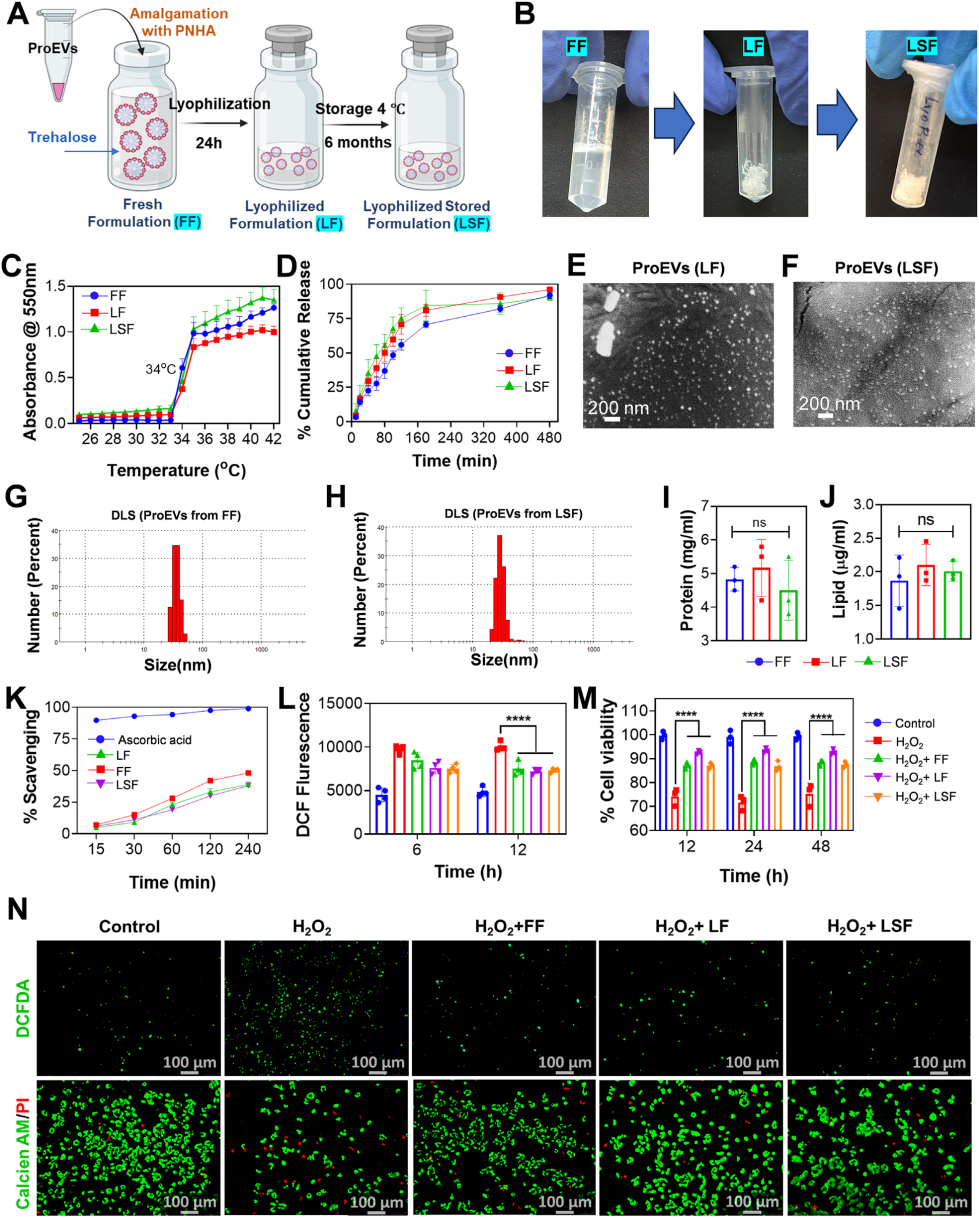
Off‐the‐Shelf therapeutic potential of PNHA‐ProEVs. (A) Graphical depiction of lyophilization process of PNHA‐ProEVs, (B) representative digital images of FF, LF and LSF, (C) LCST behavior of FF, LF and LSF showing no significant alteration in LCST, (D) ProEVs release kinetics from FF, LF and LSF, (E) and (F) Scanning electron microscopy images of released ProEVs from LF and LSF, (G) and (H) dynamic light scattering analysis of ProEVs from LF and LSF, (I) and (J) protein and lipid content estimation of ProEVs released from FF, LF and LSF, (K) DPPH assay data plot showing comparable anti‐oxidant potential of FF, LF and LSF, (L) DSF‐DA data plot showing reduced DCF fluorescence in H_2_O_2_ challenged cells post FF, LF and LSF treatment, (M) MTT assay data plot showing improved cell survival of H_2_O_2_ challenged cells post FF, LF and LSF treatment, (N) Upper panels show fluorescence images of DCF‐DA‐labeled cells indicating oxidative stress modulation, and Calcein‐AM/PI staining (bottom row) shows increased live cell population post‐treatment.

## 3 Conclusion

This study introduces a promising, cell‐free therapeutic strategy for IBD, combining probiotic‐derived extracellular vesicles (ProEVs) with a thermo‐responsive PNHA copolymer matrix for localized, endoscopically guided rectal delivery. This smart biomaterial system significantly outperforms conventional oral and rectal VSL#3 formulations in mitigating inflammation, restoring epithelial barrier integrity, and rebalancing the gut microbiota in a murine model of colitis. This hybrid platform effectively suppresses inflammatory mediators such as TNF‐α and fecal calprotectin, while promoting the expression of key barrier and regenerative markers. It facilitates macrophage repolarization toward an anti‐inflammatory M2 phenotype. Multi‐omics analyses underscore the broad‐spectrum impact of ProEVs in reprogramming host transcriptomic and proteomic landscapes toward tissue repair, immune resolution, and microbial homeostasis. This work likely represents the first demonstration of a biomaterial‐encapsulated, rectally delivered probiotic‐derived vesicle therapy that simultaneously addresses three critical pillars of IBD pathology: immune dysregulation, epithelial damage, and microbiota imbalance. The non‐cellular, localized delivery approach offers enhanced safety, stability, and translational feasibility compared to live probiotics. While the study is limited to a preclinical model of acute colitis, future investigations in chronic and relapsing models will be essential to evaluate long‐term efficacy, durability of response, and progression toward clinical validation. Together, our findings lay the groundwork for a next‐generation, multifunctional, and clinically adaptable therapy for IBD, opening new avenues in the field of smart biomaterials and microbiome‐based interventions.

## 4 Experimental Section/Methods

### 4.1 Materials Used in the Study

Sun Pharma's VSL#3 probiotic capsules were purchased from a nearby vendor. *N‐tert*‐Butyl acrylamide (NTBAM), N,N,N', N'‐Tetramethylethylenediamine (TEMED), ammonium persulfate (APS), *N‐*hydroxysuccinimide (NHS), sodium salt of hyaluronic acid from *Streptococcus equi* (HA), 4′,6‐diamidino‐2‐phenylindole (DAPI), 3‐(4,5‐dimethylthiazol‐2‐yl)‐2,5‐diphenyltetrazolium bromide (MTT), lipopolysaccharide from *Escherichia coli* (LPS), and Dulbecco's modified Eagle's medium (DMEM) were obtained from Sigma–Aldrich. Wako Pure Chemical Company (Japan) provided *N‐*Isopropylacrylamide (NIPAM). Bovine serum albumin, sodium citrate dihydrate, *N‐*1‐naphthylethylenediamine dihydrochloride, 1‐ethyl‐3‐(3‐(dimethylamino)propyl) carbodiimide (EDC), 2‐aminoethanethiol hydrochloride (AET·HCl), sulfanilamide, Titanium(IV) sulfate, and ethylenediaminetetraacetic acid (EDTA) were provided by Loba Chemie (Mumbai, India). Merck supplied potassium persulfate (KPS), and TCI Chemicals (Tokyo, Japan) supplied 5‐aminosalicylic acid (5‐ASA). Fetal bovine serum (FBS) and RPMI‐1640 medium were supplied by Gibco. Agar powder, agarose, *Lactobacillus* MRS (De Man, Rogosa and Sharpe) broth medium, and antibiotic‐antimycotic solution (P/S) 100X were provided by Himedia (India). Life Technologies (Abcam) provided the goat anti‐mouse secondary antibody and phalloidin Alexa Fluor‐488 conjugated. The rat ZO‐1 antibody (ZMD.437) was purchased from Invitrogen, and the Alexa Fluor‐488 conjugated goat anti‐rabbit secondary antibody was purchased from Jackson Immuno Research. The antibodies against CD163 and iNOS were procured from Santa Cruz Biotechnology.

### 4.2 Synthesis and Characterization of Hyaluronic Acid Conjugated Poly(*N‐*Isopropyl Acrylamide‐*co*‐Tertbutyl Acrylamide) (PNHA)

PNIPAM and HA copolymer was synthesized by our well‐established procedure [29]. In a nutshell, 40 mL of degassed water was used to dissolve NIPAM and NTBAM monomers, and the solution was kept at 29°C in a water bath. Then, to create P(NIPAM*‐co‐*NTBAM)‐NH_2_ (PNIPAM with terminal amine functionalization), 0.005 M KPS (initiator) and 0.01 M AET·HCl (chain transfer agent) were dissolved in distilled water (10 mL) and added to the reaction mixture. With magnetic stirring, the polymerization reaction was carried out for 6 h in a nitrogen atmosphere. After dialysis against deionized water, the resultant product was purified and freeze‐dried. P(NIPAM*‐co‐*NTBAM)‐NH_2_ and HA were dissolved in deionized water at a concentration of 0.5% w/v and a feed ratio of 70:30 at room temperature (25°C) for the purpose of graft copolymerization. 0.1 M HCl was used to bring the mixture's pH down to about 5. The reaction was uniformly stirred for 24 h while the NHS and EDC were added to maintain a HA/EDC/NHS molar ratio of 2:2:1. The finished product (PNHA) was freeze‐dried after being purified by dialysis against distilled water. The successful copolymerization was confirmed by Fourier transform infrared spectroscopy (FTIR) using the conventional KBr pellet method. The absorbance of 0.1% w/v aqueous polymer solutions was measured at 540 nm across temperatures from 25 to 50°C using a UV–vis spectroscope (Cary 60 UV–vis, Agilent Technologies) equipped with a temperature control device (Cary Single Cell Peltier Accessory) to measure the LCST of PNHA, with or without ProEVs. An MCR102 rheometer (Anton Paar) with parallel‐plate geometry, a cryostat, and a temperature controller were used to analyze the rheological characteristics of the polymer formulations. In order to investigate the effect of temperature on loss modulus (G″), storage modulus (G′), and complex viscosity (η), temperature sweep measurements were performed in the temperature range of 15–50°C at a rate of 1.0°C/min. The linear viscoelastic region was identified by amplitude sweeps at the LCST at an angular frequency of 1 rad/s and a shear strain ramp of 0.1%–100%.

### 4.3 Ex‐Vivo Adhesion and Retention Studies

Freshly excised rat colon explants were thoroughly washed with phosphate‐buffered saline (PBS) and placed on sterile glass slides. The lyophilized PNHA formulation was reconstituted in water (2.5% w/v) and uniformly applied onto the mucosal surface of the tissue, followed by incubation at 37°C to allow sol‐gel transition. To evaluate colonic retention and degradation behavior, PNHA‐coated explants were immersed in simulated intestinal fluid (SIF, pH 6.8) and maintained at 37°C under gentle agitation. At predetermined time points (0, 24, 48, and 72 h), the explants were removed, and the residual polymer adhering to the tissue was carefully recovered by washing with cold water into pre‐weighed tubes. The collected residues were subsequently frozen, lyophilized, and weighed to determine polymer retention. Colonic retention of the polymer was calculated using the following formula:

(1) $$\%\;\text{Retention at time}\;\;t=(W_{t}/W_{0})\times 100$$

Where: W_0_ = weight at 0 h (initial dry weight), W_t_ = weight at different time points. Result is expressed as percentage of material retained.

### 4.4 Culturing of VSL#3 Strains and Isolation of Genomic DNA

VSL#3 organisms were inoculated into MRS broth medium under anaerobic conditions and incubated at 37°C for 24 h. When the OD reached 1, the cultures were centrifuged at 4,000 x g for 15 min to pellet the bacterial cells. To confirm the presence of all the bacterial strains in the culture, genomic DNA was isolated from the pelleted cells by a previously established protocol [68] to confirm the presence of all bacterial strains present in the VSL#3 probiotic blend, PCR was performed targeting the DNA gyrase B (*gyrB*) gene, a conserved housekeeping gene, for each strain. The PCR products were run on a 2% agarose gel, and the presence of specific DNA bands for each bacterial strain was verified under UV light. The primer sequences used in the PCR are given in Table S1.

### 4.5 ProEVs Isolation and Characterization

ProEVs were extracted from the VSL#3 probiotic organism broth culture. For this, the organisms were cultivated for around 24 h at 37°C in *Lactobacillus* MRS broth medium in anaerobic conditions. When the OD reached 1, the bacterial cells and debris were removed by centrifugation for 20 min at 9000×g, the supernatant was filtered through 0.22 µm filters. By using an Amicon 100 kDa filter for ultrafiltration, the exosome‐enriched medium was collected. The concentrate underwent 2 h of ultracentrifugation at 150 000×g. After centrifuging the precipitate once more for an hour at 150 000×g, it was resuspended in sterile PBS and stored at −80°C until it was needed, which was one month of isolation. Numerous methods, such as dynamic light scattering (DLS), atomic force microscopy (AFM), nanoparticle tracking analysis (NTA), and transmission electron microscopy (TEM) were used to characterize the isolated ProEVs. The ProEVs were dried on a glass coverslip, fixed in 4% paraformaldehyde for 15 min, and imaged at an accelerating voltage of 10 kV for FESEM analysis. ProEVs were imaged at an accelerating voltage of 120 kV for TEM analysis after being fixed in 1% glutaraldehyde on a formvar/carbon grid. The ProEVs suspension was diluted to 1 mL in Milli‐Q water for size distribution and zeta potential analysis, and a Malvern Zetasizer ZS90 was utilized for the analysis. Particle Metrix‐ZetaView system was used for NTA and zeta potential measurement.

### 4.6 ProEVs Encapsulation and Release From the Copolymer

The ProEVs‐loaded polymer system (PNHA‐ProEVs) was prepared by combining the ProEVs suspension with PNHA. Specifically, 2.5% PNHA was dissolved in sterile 0.1 M PBS and then mixed with 100 µg of ProEVs. This mixture was maintained at 4 °C for 1 h with gentle stirring. The polymer formulation was then transferred into centrifuge tubes and incubated at 37 °C to induce gelation. Subsequently, 200 µL of PBS was added as the release medium over the gelled polymer. At designated time points, 75 µL of PBS was collected to measure the protein content of the released exosomes using a BCA assay. At each time point, the collected solution was replaced by fresh PBS. Similarly, for the 5‐ASA release study, 1 mg of the drug was encapsulated within the polymer matrix, and the release profile was monitored at different time points using UV–vis spectroscopy at 330 nm wavelength.

### 4.7 Quantification of Protein and Lipid Content of ProEVs

Protein content of the ProEVs was quantified using a BCA assay by following the manufacturer's protocol. Lipid content of ProEVs was quantified by modified sulfophosphovanillin (SPV) assay. Briefly, Lipid standard solution was prepared in chloroform (2 mg/mL) comprising of cholesterol and potassium oleate. After that, 70 µL of standards were heated at 90°C for 10 min for chloroform evaporation. 50 µL exosome suspension (or PBS for standards) was then added, followed by 250 µL of 96% H_2_SO_4._ The mixture was heated at 90°C for 20 min, and 220 µL was added to a 96‐well plate to cool down to room temperature. Vanillin (110 µL of 0.2 mg/mL) in 17% phosphoric acid was then added to each well and incubated for 10 min at room temperature. Finally, 200 µL of the solution was taken, and absorbance was measured at 540 nm. For protein and lipid estimation of ProEVs released from the polymer matrix, the complete release medium was first subjected to ultracentrifugation, following which the obtained pellet was resuspended in sterile PBS for further analysis.

### 4.8 Antioxidant Assay

A DPPH assay was used to assess the antioxidant qualities of PNHA and PNHA‐ProEVs. In this assay, 200 µL of 200 µm DPPH solution in ethanol at 37°C was incubated with 100 µL of 2.5% w/v PNHA alone and PNHA‐ProEVs. Over the course of 240 min, the absorbance at 517 nm was observed in relation to the control DPPH solution. The antioxidant capacity was assessed by calculating the percentage inhibition using the following formula:

$$\%\;\text{ROS scavenging}=\frac{\left(\text{AB}-\text{AS}\right)}{\text{AB}}\times 100$$

where AS is the absorbance of the sample (DPPH+PNHA or PNHA‐ProEVs) and AB is the absorbance of the control (DPPH only).

For H_2_O_2_ scavenging, 10 mm H_2_O_2_ was mixed with different samples (PNHA, PNHA‐ProEVs) in 2 mL of PBS (pH 7.4). 50 µL of the above solution was mixed with 100 µL of 13.9 mm Ti(SO_4_)_2,_ and the absorbance at 405 nm was observed every 10 min until 240 min.

### 4.9 Cell Culture

The National Centre for Cell Science provided the HT‐29 colonic epithelial cell line and Raw 264.7 murine macrophages (NCCS, Pune, India). Both cell lines were cultivated in DMEM medium containing 10% v/v FBS and 1% v/v penicillin/streptomycin and incubated at 37°C in a humidified environment with 5% CO_2_.

### 4.10 Cytocompatibility and Cell Survival Studies

For cytocompatibility assessments, HT‐29 cells were seeded at a density of 10^4^ cells per well in 96‐well plates. After a 24‐h of incubation, the cells were exposed to ProEVs, PNHA, and PNHA‐ProEVs, and cell viability was evaluated at various time intervals. In cell proliferation experiments, 5 × 10^3^ cells were seeded per well in 96‐well plates and treated after 24 h of incubation, with cell viability monitored for up to 7 days. Additionally, the impact of ProEVs alone and PNHA‐ProEVs on cell survival under TNBS‐induced stress was investigated using HT‐29 cells cultured in 96‐well plates. After cell attachment, the cells were pre‐treated with exosomes (20 µg/mL) and/or 2.5% w/v PNHA for 12 h before exposure to 300 µg/mL TNBS in basal DMEM media. Some groups received TNBS pretreatment followed by ProEVs, PNHA, and PNHA‐ProEVs. Cells exposed to TNBS stress without exosome pre‐treatment served as the negative control, while cells in basal media were the positive control. Metabolic activity was assessed over 72 h using the MTT assay.

### 4.11 In Vitro Antioxidant Potential of PNHA‐ProEVs

The antioxidative potential of PNHA‐ProEVs was investigated using HT‐29 and RAW264.7 cell lines. Initially, 2 × 10^5^ cells were seeded in a 24‐well plate and treated with H_2_O_2_ (1 mm). After a 30 min incubation, the H_2_O_2_‐containing medium was changed, and fresh cell culture medium was added. The cells were subsequently treated with either ProEVs with or without PNHA. After 12 h, the cells were washed and exposed to DCFH‐DA (10 µm) for fluorescence analysis of oxidative stress, which was measured at excitation/emission wavelengths of 485/535 nm after 1, 2, and 3 h. Furthermore, the cells were washed with PBS twice and examined under a fluorescence microscope. Furthermore, the impact of ProEVs and/or PNHA on cell survival under oxidative stress was evaluated using HT‐29 cells. The cells were cultured in 96‐multiwell plates, and after attachment, they were pre‐treated with exosomes (20 µg/mL) and/or 2.5% w/v PNHA for 12 h before exposure to oxidative stress induced by 200 mm H_2_O_2_ in basal media. Cells exposed to oxidative stress without exosome pre‐treatment served as the negative control, while cells in basal media acted as the positive control. Metabolic activity was monitored for 24 h at regular intervals using the MTT assay as previously described.

### 4.12 In Vitro Confocal Microscopy

To explore the uptake of ProEVs by HT‐29 cells, the cells were initially seeded onto coverslips in a 24‐well plate containing culture medium (5 × 103 cells/well). Following this, ProEVs at a concentration of 20 µg/mL were mixed with Calcein AM (10 µm) and incubated together at 37°C for 20 min. Subsequently, any unbound dye was removed by centrifuging the ProEVs and washing them with PBS using a 100 kDa filter (Amicon). The ProEVs labeled with Calcein AM were then introduced to the cells, and after 3 h of incubation, images were captured using confocal microscopy (Leica SPII, Germany). For studies on morphology and viability, HT‐29 cells were seeded in culture medium onto 24‐well plates (5 × 10^3^ cells/well). Following various treatments and incubation periods, the cells underwent three washes with 1X PBS and were stained with Calcein AM (1:1000) and PI (1:1000) for live/dead cell visualization. For morphological analysis, the cells were washed, fixed using 4% paraformaldehyde, and permeabilized with Triton X‐100. They were subsequently treated with Alexa Fluor 488 phalloidin to label F‐actin filaments, while DAPI was utilized as a counterstain to highlight the cell nuclei. The cellular nuclei and cytoskeleton were examined using a confocal microscope (Leica SPII, Germany).

### 4.13 In Vitro Assessment of Immunomodulatory Properties

To assess iNOS and CD163 levels in RAW 264.7 murine macrophages, cells were seeded onto 24‐well plates containing culture medium (5 × 10^4^ cells/well). Following a 24 h incubation, cells were exposed to either 10 µg/mL of LPS or control medium for 12 h. Subsequently, the cells received treatments of ProEVs or PNHA‐ProEVs formulations for an additional 24 h. The cells were then fixed and permeabilized using 4% PFA for 10 min followed by 0.1% Triton X‐100 for another 10 min. After blocking with PBST containing 1% BSA and 10% goat serum for 2 h, the cells were stained with anti‐iNOS and anti‐CD163 (mouse reactivity) rabbit polyclonal antibodies (1:400). This was followed by incubation with anti‐rabbit IgG secondary antibodies conjugated with AlexaFluor‐488 and AlexaFluor‐555, and counter‐staining with DAPI. Confocal laser‐scanning microscopy (Leica Germany) was then used to capture images from random fields of view. For quantifying the nitric oxide produced by LPS‐stimulated murine macrophages (RAW 264.7), the stable nitric oxide conversion product, nitrite (NO_2_), was measured using the Griess‐Saville method. RAW 264.7 cells were exposed to LPS (10 µg/mL in basal DMEM) for 12 h. Following LPS stimulation, cells were treated with ProEVs or PNHA‐ProEVs formulations (ProEVs at 20 µg/mL, PNHA at 2.5% w/v) for 12 h, followed by incubation in phenol red‐free basal DMEM medium for an additional 24 h. Subsequently, 100 µL of spent media from each sample well was collected and combined with an equal volume of Griess reagent, prepared by mixing 1% sulfanilamide and 0.1% N‐(1‐naphthyl)‐ethylene diamine hydrochloride in 2.5% H_3_PO_4_. After a 15 min incubation, absorbance at 540 nm was measured using a UV–vis microplate reader. A sodium nitrite standard curve was utilized as a reference for calculating the amount of nitrite present.

### 4.14 Flow Cytometric Analysis of Macrophage Polarization

To evaluate the effect of PNHA, ProEVs, and PNHA‐ProEVs on macrophage polarization, RAW 264.7 murine macrophages were seeded in 12‐well plates at a density of 1 × 10^5^ cells/well and allowed to adhere overnight. Upon reaching ∼60% confluence, cells were stimulated with LPS (10 µg/mL) for 6 h, followed by treatment with PNHA, ProEVs, or PNHA‐ProEVs, while untreated cells served as controls (n = 3 wells/group). After 12 h of treatment, media were replaced with basal medium, and cells were further incubated for 24 h

Following treatment, cells were harvested in cold PBS, pooled from three wells per group, centrifuged at 400 × g for 5 min at 4°C, and resuspended in FACS buffer. To reduce nonspecific binding, cells were incubated in 10% FBS‐containing buffer for 15 min at 4°C and subsequently stained with FITC‐conjugated anti‐CD86 (BioLegend) and APC‐conjugated anti‐CD206 (Cell Signaling Technology) antibodies for 30 min at 4°C in the dark. After washing, cells were resuspended in FACS buffer containing propidium iodide (PI; 1 µg/mL) to exclude dead cells

Flow cytometry was performed using a BD FACSMelody cell sorter, and 50 000 events were acquired per sample. Live singlet macrophage populations were analyzed for CD86 and CD206 expression using FlowJo v11 software.

### 4.15 Animals

All animal experiments were performed according to the guidelines set forth by the Institutional Animal Ethics Committee (IAEC) at the Indian Institute of Technology Kanpur (IAEC Protocol No. IITK/IAEC/2021/1121, IITK/IAEC/2025/1265 and IITK/IAEC/2024/1212) and were approved by the same. The animals used in this study were sourced from CSIR‐Central Drug Research Institute (Lucknow, Uttar Pradesh, India) and were a mix of littermates. They were housed under controlled and clean conditions within the animal facility at IIT Kanpur. To ensure consistency in the gut microbiota among the rats and minimize heterogeneity, the rats were co‐housed for one week prior to being randomly assigned to the experimental groups. During the experiments and outcome assessments, the investigators were not blinded to the allocation, unless specified otherwise in specific sections that required blind assessment.

### 4.16 In Vivo Long‐Term Biocompatibility Assessment

The in vivo biocompatibility of the PNHA formulation was initially evaluated by implantation into dorsal muscle pouches of 6‐week‐old Wistar rats, following Institutional Animal Ethics Committee guidelines (IITK/IAEC/2021/1121). Animals were anesthetized using isoflurane (2–4%), and sterile surgical procedures were performed to create dorsal muscle pockets, into which 0.5 mL of 2.5% (w/v) PNHA solution was injected. Control animals received PBS. The incision sites were sutured, and animals were monitored over two weeks for behavioral changes and body weight variations. Blood samples were collected at defined intervals for hematological analysis, and serum biochemical parameters were assessed using a semi‐automated analyzer. Upon completion, major organs including liver, lungs, heart, kidneys, thymus, spleen, and colon were harvested, fixed, and processed for histological evaluation using H&E staining. All histological analyses were performed in a blinded manner. In addition to implantation‐based assessment, we further evaluated the safety of PNHA under clinically relevant administration conditions by performing a 15‐day repeated rectal dosing study. Following daily intracolonic administration, animals were assessed for body weight changes, hematological and biochemical parameters, and colonic histology. Consistent with the implantation study, no signs of systemic toxicity, local inflammation, or tissue damage were observed, confirming the excellent biocompatibility and safety of the formulation upon repeated local administration.

### 4.17 In Vivo Colitis Model

Female Wistar rats aged six weeks were accommodated in cages with four rats per cage and allowed to acclimatize for one week before being included in the study. The rats were divided into six groups, each containing eight animals. To induce colitis, a combination of 40% alcohol and TNBS at a dose of 100 mg/kg was administered intra‐rectally to the rats, 8 cm from the rectum, for seven consecutive days. Healthy control rats were given PBS instead. On the eighth day, the rats underwent bowel cleansing with oral laxatives, followed by the intrarectal administration of PNHA, PNHA‐5ASA, PNHA‐ProEV, PNHA‐5ASA‐ProEV, or PBS, as per the schedule outlined in Table S2. Changes in body weight and disease activity were monitored daily for a period of 21 days. Blood samples were collected at predetermined time points from the venous sinus. On the final day of the experiment, fecal samples were gathered for microbiota analysis, and the rats were euthanized. The entire colon was removed, and its length was measured after rinsing with PBS. Four distal colon segments were preserved for histological examination, while another four were designated for gene expression studies.

### 4.18 Disease Activity Index (DAI) Evaluation

The Disease Activity Index (DAI) was evaluated by observing changes in stool consistency, body weight, and rectal bleeding, using an established protocol [61] as a guide. Briefly, alterations in stool consistency, body weight, and rectal bleeding were monitored daily to determine the DAI. Each parameter was assigned a score based on the following criteria: body weight loss (0 for no loss, 1 for 1–5%, 2 for 5–10%, 3 for 11–15%, 4 for >15%), diarrhea severity (0 for normal, 2 for loose stools, 4 for watery diarrhea), and intensity of rectal bleeding (0 for normal, 2 for slight bleeding, 4 for severe bleeding). The DAI score was computed as the average of these individual scores.

### 4.19 In Vivo Enzyme‐Linked Immunosorbent Assay (ELISA) Analysis

For determination of TNF‐α and calprotectin levels in rat serum, the blood samples were withdrawn from rats at different points, and serum was separated by centrifugation at 2000–3000 RPM for 15–20 min at 4°C. The concentration of these two biochemical markers was measured by ELISA kits (KRISHGEN Biosystems) according to the manufacturer's protocol.

### 4.20 FITC‐Dextran Translocation Experiment

Intestinal permeability was assessed using a fluorescein isothiocyanate‐dextran (FITC‐dextran) assay. At the end of the treatment period, animals were fasted for 4–6 h with free access to water. FITC‐dextran (average molecular weight ∼4 kDa) was prepared in sterile PBS and administered via oral gavage at a dose of 600 mg/kg body weight. After 4 h of administration, blood samples were collected via the retro‐orbital route, and serum was isolated by centrifugation at 3000 rpm for 10 min. The fluorescence intensity of FITC‐dextran in serum was measured using a fluorescence microplate reader (excitation: 485 nm; emission: 528 nm). A standard calibration curve of FITC‐dextran in serum was used to quantify the concentration, and results were expressed as µg/mL of FITC‐dextran in serum, reflecting intestinal permeability.

### 4.21 Histology

For histological studies, 1 cm long segment of the distal colon was fixed with 4% (v/v) neutral buffered formalin and kept in 70% alcohol and then embedded in paraffin. Tissue sections of 7–8 µm thickness were obtained and stained with hematoxylin and eosin (H&E) and alcian blue and periodic acid‐Schiff (PAS). A slide scanner (Morphle Labs) was used to obtain the images.

### 4.22 In Vivo Immune‐Fluorescence Imaging

Tissue sections were washed with PBS and rehydrated in order to produce in vivo confocal microscopy images. After permeabilization with 0.1% Triton X‐100 for 10 min, antigen retrieval was performed using heated sodium citrate buffer at 95°C. Following this, the tissue sections were blocked with a solution of 1% BSA and 10% goat serum in PBST (PBS + 0.1% Tween 20). They were then stained with the following specific antibodies: ZO‐1 (Invitrogen, 1:400) for an overnight period at 4°C; goat anti‐rabbit Alexa Fluor‐488 secondary antibody (Jackson Immuno Research; 1:400) for 2 h at room temperature; iNOS (Santa Cruz Biotechnology) followed by goat anti‐mouse Alexa Fluor‐555 secondary antibody (Invitrogen); and CD163 (Santa Cruz Biotechnology) followed by goat anti‐mouse Alexa Fluor‐488 secondary antibody (Abcam). Lastly, DAPI was used as a counterstain for the nuclei. Random fields of view were imaged using the Leica SPII, Germany confocal laser scanning microscope.

### 4.23 Quantitative Reverse Transcription PCR

Using the TRI reagent, RNA was extracted from RAW 264.7 cells for the in vitro study and colon tissues for the in vivo study in order to perform qPCR investigations. The High‐Capacity cDNA Reverse Transcription Kit from Applied Biosystems was then used to perform reverse transcription. Gene expression was quantified using the SYBR Green qPCR kit, which is also available from Applied Biosystems. The PCR cycling conditions included a 3 min initial denaturation at 95°C, 40 cycles of denaturation at 95°C for 15 sec, and 1 min of annealing/extension at 60°C. RPL32 was used as the reference gene in order to calculate the target genes' relative expression levels. Tables S3 and S4 provide information on the primer sets that were used for amplification.

### 4.24 Microbiota Analysis

To analyze the microbiota, fresh fecal samples were gathered in sterile containers, carefully packaged, and sent to Biokart India Pvt. Ltd. in Bengaluru, Karnataka (India). Before starting the PCR amplification process, the genomic DNA was extracted using the Xploregen kit, and its quality was evaluated using gel and NanoDrop electrophoresis. Each sample's amplicons were purified using Ampure beads to get rid of any leftover primers after amplification. To prepare the sequencing libraries, eight more PCR cycles were carried out using Illumina barcoded adapters. Following another purification with Ampure beads, these libraries were measured using the Qubit dsDNA High Sensitivity assay kit. The 2 × 300PE v3‐v4 sequencing kit and the Illumina Miseq platform were used for the sequencing process. The NCBI database was used for bioinformatics analysis of the 16s V3‐V4 regions. Fastq raw data files were created by demultiplexing the bcl data that was acquired from the sequencer. The tools Multiqc (Version 1.10.1) and Fastqc (Version 0.11.9) were used to evaluate the quality of the demultiplexed data. Microsoft Excel (2016) was used to create abundance feature tables and identify the top ten organisms in each sample. Microbiota Analyst 2.0 was used to perform additional analyses, including heatmap, core microbiota, alpha and beta diversity, and rarefaction curve.

### 4.25 Proteomics Profiling of ProEVs

Freshly isolated ProEVs were suspended in sterile PBS and sent to Biokart India Pvt. Ltd. (Bangalore, India) for performing LC‐MS/MS and data analysis. Briefly, Extracellular vesicle (EV) pellets were washed with MS‐grade water and sequentially extracted using lysis buffers. The pooled supernatant was acetone‐precipitated overnight, and the resulting pellet was dissolved in 50 mm ammonium bicarbonate with 0.1% SDS. For proteomic profiling, 100 µg of protein was reduced with 100 mm DTT (95°C, 1 h) and alkylated with 250 mm iodoacetamide (IDA) in the dark for 45 min. Trypsin digestion was performed overnight at 37°C, followed by vacuum drying and reconstitution in 0.1% formic acid. Peptides were desalted, centrifuged, and 10 µL of the sample was injected into a UPLC BEH C18 column (40°C, 0.3 mL/min) using a water–ACN gradient containing 0.1% formic acid. Eluted peptides were analyzed by ESI‐QTOF LC‐MS/MS. Raw data were processed using MassLynx 4.1 and peptide identification was performed using Progenesis software.

### 4.26 Transcriptomic Profiling of Colon Tissue

Post‐sacrifice, colon tissue was harvested from healthy, diseased, and PNHA‐ProEVs‐treated animals and sent to Biokart India Pvt. Ltd. (Bangalore, India) for transcriptomic analysis. Briefly, total RNA was extracted and subjected to quality control (QC) using standard integrity and concentration assessments. High‐quality RNA samples were fragmented and processed for library preparation, followed by QC of the prepared libraries. Sequencing was performed using the Illumina platform with 150 bp paired‐end reads. Raw sequencing data underwent quality assessment and trimming to remove low‐quality bases and adapters. High‐quality reads were then used for downstream analyses, including alignment, quantification, and differential expression analysis.

### 4.27 Lyophilization and Cold Storage of PNHA‐ProEVs

A 2.5% PNHA copolymer solution was prepared in PBS and loaded with 100 µg/mL ProEVs, followed by the addition of trehalose (2%) as a cryoprotectant. The formulation was frozen at ‐80°C and lyophilized to yield a stable white flaky matrix. One batch was analyzed immediately after lyophilization, while a parallel batch was stored at 4°C under aseptic conditions for six months before rehydration.

### 4.28 Statistical Analysis

To guarantee reliability, every experiment was carried out a minimum of twice using duplicate measurements. The findings are shown as means (SD) with standard deviations. Tukey's HSD multiple comparison post hoc test was performed after one of two ANOVA methods to evaluate differences between the groups. The data revealed comparable variance among the groups and an approximate normal distribution. As stated in the figure captions, experiments were independently repeated several times. The entire set of data from a single independent experiment is represented by the dataset shown in the figure. In the analysis, no samples were disqualified. ^*^ *p* < 0.05, ^**^ *p* < 0.01, ^***^ *p* < 0.001, and ^****^ *p* < 0.0001 denote statistical significance. With GraphPad Prism 9.0 (GraphPad Software, La Jolla, CA), statistical analyses were performed.

## Author Contributions

**Jan Marsal**: writing – review and editing, validation. **Ubaid Tariq**: methodology, data curation, formal analysis, validation. **Shreya Mehrotra**: formal analysis, visualization, validation. **Saravanan Matheshwaran**: validation, formal analysis, visualization. **Ashok Kumar**: conceptualization, supervision, funding acquisition, project administration, resources, writing – review and editing, validation. **Ayushi Mairal**: conceptualization, methodology, software, data curation, investigation, validation, writing – original draft, formal analysis, visualization, writing – review and editing.

## Conflicts of Interest

The authors declare no conflicts of interest.

## Supporting information




**Supporting File 1**: advs76585‐sup‐0001‐SuppMat.docx.

**Supporting File 2**: advs76585‐sup‐0002‐Supporting MovieS1.mp4.

**Supporting File 3**: advs76585‐sup‐0003‐Supporting MovieS2.mp4.

## Data Availability

The data that support the findings of this study are available from the corresponding author upon reasonable request. All relevant data are included within the article and its Supporting Information.
