# Supplementary material for: Probiotic‐Derived Vesicles Rectally Delivered via Thermo‐Gelling Copolymer Promote Mucosal Healing and Reduce Inflammation in Ulcerative Colitis
Source: Adv Sci (Weinh). 2026 Jul 17:e76585. Online ahead of print. doi: 10.1002/advs.76585 (PMC13379261; doi:10.1002/advs.76585)
Supplement: Supplementary file 1 — Supporting File 1: advs76585‐sup‐0001‐SuppMat.docx. [file ADVS-9999-e76585-s001.docx]

Supporting Information

**Probiotic-derived vesicles rectally delivered via thermo-gelling copolymer promote mucosal healing and reduce inflammation in ulcerative colitis**

Ayushi Mairal^1^, Ubaid Tariq^1^, Shreya Mehrotra^1^, Saravanan Matheshwaran^1,2,3^, Jan Marsal^4^ and Ashok Kumar^1,2,3,5,6*^

1. *Department of Biological Sciences and Bioengineering; Indian Institute of Technology Kanpur, Kanpur 208016, UP, India*
2. *Centre for Environmental Science and Engineering, Indian Institute of Technology Kanpur, Kanpur 208016, UP, India*
3. *The Mehta Family Centre for Engineering in Medicine, Indian Institute of Technology Kanpur, Kanpur 208016, UP, India*
4. *Department of Clinical Sciences, Lund University and Skåne University Hospital, SE-22100, Lund, Sweden*
5. *Centre of Excellence for Materials in Medicine, Gangwal School of Medical Sciences and Technology, Indian Institute of Technology Kanpur, Kanpur-208016, UP, India*
6. *Centre for nanosciences, Indian Institute of Technology Kanpur, Kanpur-208016, UP, India*

*Correspondence should be addressed to

**Prof. Ashok Kumar**

Department of Biological Sciences and Bioengineering
Indian Institute of Technology Kanpur,
Kanpur-208016, UP, India
Email: [ashokkum@iitk.ac.in](mailto:ashokkum@iitk.ac.in)
Phone:+91-512-2594051

**Figure S1.** **(A)** Reaction schematics showing two-step synthesis procedure of PNHA, **(B)** FTIR Spectra of NIPAM monomer, PNIPAM and PNHA, **(C)** F-actin and DAPI staining images of HT29 cells showing no changes in cellular morphology post PNHA-ProEVs treatment, **(D)** % Cumulative release of 5-ASA from PNHA formulation at different timepoints up to 24h **(E-F)** Data plot and digital images of haemolysis assay of ProEVs with rat blood showing good hemocompatibility of ProEVs.

**Figure S2.** **(A)** Representative digital image of PNHA application of PNHA on luminal surface of rat colon explant at room temperature, **(B)** Sol-gel transition of PNHA layer at 37 ℃ temperature, **(C)** Stable PNHA layer at 90^o^ angle, **(D)** and **(E)** Stable PNHA layer can be seen on colon explant under water at 37 ℃, **(F)** PNHA degradation on colon tissue under simulated intestinal condition at different timepoints, **(G)** % Colonic retention of the polymer on the colon tissue at different time intervals.


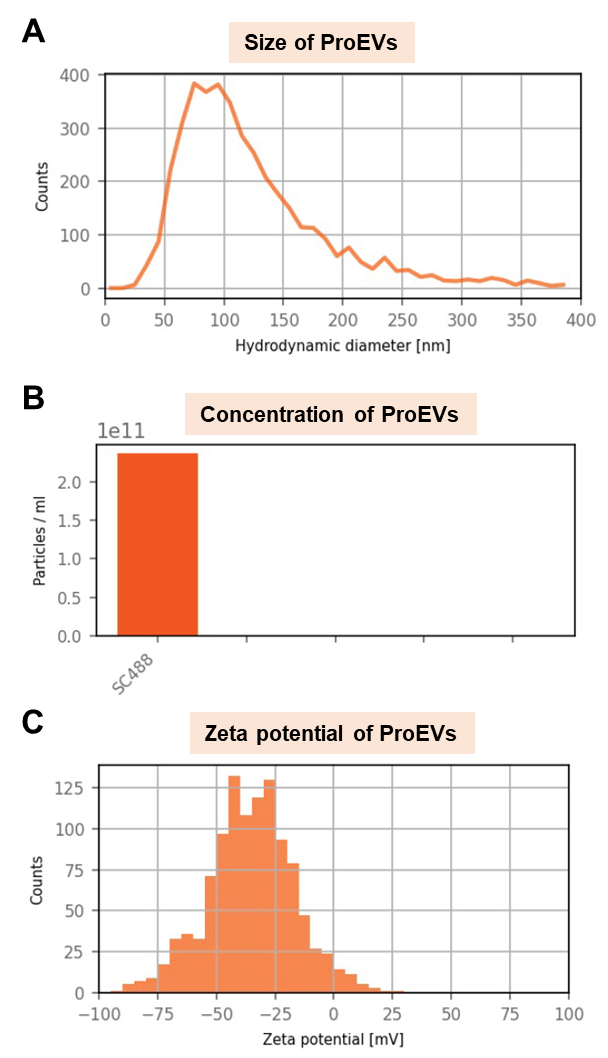


**Figure S3. (A)** Nano particle tracking (NTA) analysis of ProEVs showing Size distrbution, **(B)** Concentration and **(C)** Zetapotential of ProEVs.

**Figure S4. (A-F)** Representative flow cytometry experiment data plots showing CD86 and CD206 expression in RAW 264.7 macrophages under control, LPS-stimulated, PNHA-treated, ProEVs-treated, and PNHA-ProEVs treated conditions.

**Figure S5.** FITC-dextran permeability assay demonstrating intestinal barrier function following different treatments.

**Movie S1. Thermal gelation of PNHA-ProEVs under aqueous condition.**

<https://drive.google.com/file/d/1EgYrQe5V8KlqTNsRwi-1V8DL3SAZPBoB/view?usp=sharing>

Video shows the thermal gelation behaviour of the PNHA-ProEVs under simulated intestinal condition. The PNHA coating on the colon explant can be seen here.

**Movie S2. Demonstration of applicability of PNHA formulation during colonoscopy procedure.**

<https://drive.google.com/file/d/16Bn9x4In_hK6JzK_D51zv5rKI1FvUyPZ/view?usp=sharing>

Demonstration of simulated colonoscopic delivery at 37 ℃, showing the flow of the PNHA formulation through a 1 m silicone tube prior to collection in a glass vial.

**Table S1.** List of primers used in PCR study to confirm presence of all VSL#3 strains in coculture.

| SN | Gene name | Sequence |
| --- | --- | --- |
| 1. | *L. acidophilus g*yrA | F 5′-CAAATGGGATCGCTGTTGGT-3′  R 5′-CACGCCCCATGATAATTCCG-3′ |
| 2. | *L. plantarum g*yrA | F 5’-TTTAAGTCGCAACACCGTGG-3’  R 5’-GATTCCTTTGGCCGTACGAC-3’ |
| 3. | *L. paracasei g*yrA | F 5’-CTTCCACGCATGATGTCCTG-3’  R 5’-CGCCTTCATGCACGTTGATA-3’ |
| 4. | *S. thermophilus g*yrA | F 5’-TCTTCCCGGTCCAGACTTTC-3’  R 5’-TTTCCTGAGCCAAACGAACG-3’ |
| 5. | *B. breve g*yrA | F 5’-GATGCTGCAGGTATGGGTTG-3’  R 5’-AGAATGTACTGGGCCTGGAC-3’ |
| 6. | *B. longum g*yrA | F 5’- CGCAGCCAAATACGTGATGA-3’  R 5’- CACTACGATGGCCTTCTTGC-3’ |
| 7. | *B. delbrueckii g*yrA | F 5’-CTCGTGCTTTGCCTGATGTT-3’  R 5’-CGTGACCGTCAACTAGCATG-3’ |

Table S2. Experimental groups of the animal study.

| SN | Groups | No. of animals | Description |
| --- | --- | --- | --- |
| 1. | Group-1, Healthy control | 8 | Healthy animals without TNBS treatment |
| 2. | Group-2, Negative control | 8 | TNBS induced colitis rats provided with PBS via rectal administration |
| 3. | Group-3, PNHA | 8 | TNBS induced colitis rats provided with PNHA via rectal administration |
| 4. | Group-4, PNHA-5ASA | 8 | TNBS induced colitis rats provided with 5-ASA (1.5 gm/kg of body weight) encapsulated in PNHA formulation via rectal administration |
| 5. | Group-5, PNHA-ProEVs | 8 | TNBS induced colitis rats provided with ProEVs (100 μg) encapsulated in PNHA formulation via rectal administration |
| 6. | Group-6, PNHA-5ASA-ProEVs | 8 | TNBS induced colitis rats provided with ProEVs (100 μg) and 5-ASA (1.5 gm/kg of body weight) encapsulated in PNHA formulation via rectal administration |

**Table S3.** List of rat primers used in *in vivo* qPCR study.

| SN | Gene name | Sequence |
| --- | --- | --- |
| 1. | ZO-1 | F 5′-CCATCTTTGGACCGATTGCTG-3′  R 5′-TAATGCCCGAGCTCCGATG-3′ |
| 2. | MUC-2 | F 5’- GCCAGATCCCGAAACCA-3’  R 5’- TATAGGAGTCTCGGCAGTCA-3’ |
| 3. | TNF-α | F 5’-CGACTCTGACCCCCATTACT-3’  R 5’-CGTCTCGTGTGTTTCTGAGC-3’ |
| 4. | IL-6 | F 5’-AAGGACCAAGACCATCCAAC-3’  R 5’-ACCACAGTGAGGAATGTCCA-3’ |
| 5. | IL-1β | F 5’-TTTGAAGAAGAGCCCGTCCT-3’  R 5’-TGTCGTTGCTTGTCTCTCCT-3’ |
| 6. | IL-10 | F 5’-GTTGCCAAGCCTTGTCAGAA-3’  R 5’-GGGAGAAATCGATGACAGCG-3’ |
| 7. | TGF-β | F 5’-TGCTTCAGCTCCACAGAGAA-3’  R 5’-TACTGTGTGTCCAGGCTCCA-3’ |
| 8. | RPL32 | F 5’-AGATTCAAGGGCCAGATCC-3’  R 5’-CGATGGCTTTTCGGTTCTTA-3’ |

Table S4. List of mouse primers used in in vitro qPCR study.

| SN | Gene name | Sequence |
| --- | --- | --- |
| 1. | iNOS | F 5’-AATCTCTGCCTATCCGTCTC-3’  R 5’-CAAGCCCTCACCTACTTCCTG-3’ |
| 2. | IL-6 | F 5’-TAGTCCTTCCTACCCCAATTTCC-3’  R 5’-TTGGTCCTTAGCCACTCCTTC-3’ |
| 3. | IL-10 | F 5’-GCTCTTACTGACTGGCATGAG-3’  R 5’-CGCAGCTCTAGGAGCATGTG-3’ |
| 4. | β-actin | F 5’-TACAGCTTCACCACC-3’  R 5’-ATGCCACAGGATTTC-3’ |

**Table S5.** Experimental groups used in animal study.

| SN. | Detail | No. of animal |
| --- | --- | --- |
| 1 | Healthy Control | 8 |
| 2. | Diseased Control | 8 |
| 3. | Oral VSL#3 | 8 |
| 4. | PNHA + VSL#3 (Rectal) | 8 |
| 5. | PNHA + ProEVs (Rectal) | 8 |

**Figure S6. (A)** Venn diagram representing the number of overlapping and unique bacterial species across different experimental groups, **(B)** Simpson diversity index representing the α diversity, **(C)** Stacked bar plots illustrating the relative abundance of microbial taxa at the Phylum, **(D)** Class **(E)** Order and **(F)** Genus levels across different groups, as determined by 16S rRNA metagenomic sequencing.

**Figure S7. (A)** Stacked bar plots illustrating the relative abundance of microbial taxa at the Phylum, **(B)** Class, **(C)** Order, and **(D)** Genus levels across different groups, as determined by 16S rRNA metagenomic sequencing.

**Figure S8. (A)** Kyoto Encyclopedia of Genes and Genomes (KEGG) Pathway analysis illustration and KEGG-based functional analysis of **(B)** Healthy, **(C)** diseased, **(D)** OralVSL#3, **(E)** PNHA-VSL#3 and **(F)** PNHA-ProEVs treated groups

**Figure S9. (A) & (B)** Volcano and bar plots depicting differentially expressed genes between the Healthy and Diseased groups, **(C) & (D)** Volcano and bar plots showing gene expression differences between the Diseased and PNHA-ProEVs-treated groups, **(E) & (F)** Volcano and bar plots representing differentially expressed genes between the Healthy and PNHA+ProEVs groups.

**Figure S10. Long term in-vivo biocompatibility assessment of PNHA after daily rectal application (A)** In vivo PNHA biocompatibility assessment work plan **(B)** Digital image of rat recieving rectal enema of PNHA, **(C)** Data plot for body weight of rats on different days suggesting no negative impact of PNHA on body weight post daily rectal application, **(D)** Histological analysis(H and E) of the colon tissue post 15 days daily application of PNHA formulation, **(E)** Data plot showing total leukocyte count of rats on different days, **(F)-(L)** biochemical parameters bilirubin, albumin, creatinine, urea, triglycerides, SGOT and SGPT quantified in rat blood serum, showing no abnormalities post PNHA rectal application for 15 days.

**Table S6.** Key ProEVs-derived proteins identified by proteomics and their potential roles in IBD therapy.

| Protein Name | Normalized Abundance | Species | Potential Role in IBD Treatment |
| --- | --- | --- | --- |
| UvrABC system protein B | 93538 | *Lactiplantibacillus plantarum* | DNA repair and protection against oxidative stress, aiding gut barrier integrity. |
| UDP-N-acetylmuramyl-tripeptide synthetase | 140546 | *Lactobacillus acidophilus* | Supports bacterial cell wall synthesis, contributing to probiotic survival and gut microbiota restoration. |
| ATP synthase subunit beta | 42276 | *Streptococcus thermophilus* | Enhances energy metabolism in probiotic bacteria, supporting gut homeostasis. |
| Manganese-dependent inorganic pyrophosphatase | 63520 | *Lactobacillus helveticus & L. acidophilus* | Regulates phosphate metabolism, maintaining microbial balance in the gut. |
| N-acetylneuraminate lyase | 102103 | *Lactiplantibacillus plantarum* | Helps degrade sialic acid, potentially modulating gut microbiota and immune response. |
| Phosphoglycerate kinase | 338751 | *Lactobacillus helveticus* | Involved in glycolysis, supporting probiotic energy metabolism. |
| Beta-galactosidase LacA | 176384 | *Lactobacillus acidophilus* | Breaks down lactose, aiding digestion and gut microbiota restoration. |
| Methylenetetrahydrofolate--tRNA methyltransferase (TrmFO) | 183206 | *Lactobacillus helveticus* | Supports bacterial growth and folate metabolism, essential for mucosal healing. |
| HPr kinase/phosphorylase | 509346 | *Streptococcus thermophilus* | Regulates sugar transport, influencing gut microbiota composition. |
| Ribosomal proteins (uS2, uS3, uL2, etc.) | 309724 | *Various species* | Essential for bacterial protein synthesis, ensuring probiotic function and gut microbiota balance. |
| Histidine--tRNA ligase | 276966 | *Lacticaseibacillus paracasei* | Supports bacterial protein synthesis, maintaining probiotic activity. |
| Carbamoyl phosphate synthase | 1241681 | *Lactiplantibacillus plantarum* | Involved in nitrogen metabolism, influencing bacterial survival in the gut. |
| Transcriptional repressor NrdR | 423340 | *Lactobacillus acidophilus* | Regulates DNA synthesis, affecting bacterial growth and probiotic stability. |
| 3-hydroxyacyl-[acyl-carrier-protein] dehydratase (FabZ) | 1084932 | *Streptococcus thermophilus* | Participates in fatty acid metabolism, contributing to bacterial membrane stability. |
| Lysine--tRNA ligase | 1188778 | *Various species* | Supports bacterial protein synthesis, crucial for gut microbiota restoration. |
| Polyribonucleotide nucleotidyltransferase | 2990933 | *Streptococcus thermophilus* | Involved in RNA processing, essential for bacterial adaptation in the gut. |
| Probable phosphoketolase 1 | 20734 | *Lactiplantibacillus plantarum* | Facilitates carbohydrate metabolism, aiding probiotic survival. |
| Glutamate--tRNA ligase | 1021910 | *Lacticaseibacillus paracasei & L. plantarum* | Supports protein synthesis and bacterial viability. |
| Uracil phosphoribosyltransferase | 713279 | *Lactobacillus helveticus* | Involved in nucleotide metabolism, supporting bacterial replication. |
| DNA mismatch repair proteins (MutL, MutS) | 4629993 | *Various species* | Maintain genetic stability in probiotics, enhancing their resilience in the gut. |
| Bifunctional protein FolD | 506951 | *Lactobacillus helveticus* | Supports folate metabolism, contributing to mucosal healing. |
| Large ribosomal subunit proteins (uL3, bL9) | 106122 | *Lactobacillus helveticus* | Essential for protein synthesis in gut microbiota. |
| Glycine--tRNA ligase beta subunit | 132058 | *Streptococcus thermophilus* | Aids protein synthesis, ensuring probiotic functionality. |
| Urocanate reductase | 836168 | *Lactiplantibacillus plantarum* | Involved in histidine metabolism, potentially influencing immune modulation. |
| CTP synthase | 193153 | *Various species* | Supports nucleotide metabolism, crucial for probiotic replication and mucosal repair. |
| RNA methyltransferase (lp_1151) | 244666 | *Lactiplantibacillus plantarum* | Modifies RNA, potentially influencing probiotic gene expression. |
| Foldase protein PrsA 2 | 39634 | *Lactiplantibacillus plantarum* | Assists in protein folding, enhancing bacterial survival in the gut. |
| CLA biosynthesis dehydrogenase/reductase | 370642 | *Lactiplantibacillus plantarum* | Produces conjugated linoleic acid, which has anti-inflammatory effects. |
| Glutathione biosynthesis bifunctional protein (GshAB) | 113680 | *Streptococcus thermophilus* | Enhances antioxidant defenses, protecting against oxidative stress in IBD. |
| Glycogen synthase | 884136 | *Lactiplantibacillus plantarum* | Helps bacteria store energy, improving probiotic persistence. |
| Chaperone protein ClpB | 351309 | *Lactiplantibacillus plantarum* | Assists in protein folding under stress, ensuring probiotic stability. |
| DNA polymerase III PolC-type | 157143 | *Lactiplantibacillus plantarum* | Essential for DNA replication, supporting probiotic survival. |
| Elongation factor Tu | 52589 | *Lactobacillus acidophilus* | Aids in protein synthesis, supporting probiotic function. |
| Lactose permease | 44598 | *Lactobacillus helveticus* | Facilitates lactose metabolism, aiding digestion and microbiota restoration. |
| Urease accessory protein UreD | 645087 | *Streptococcus thermophilus* | Involved in nitrogen metabolism, potentially influencing gut microbiota balance. |
| S-layer protein | 244849 | *Lactobacillus helveticus* | May play a role in bacterial adhesion to mucosal surfaces, promoting gut barrier protection. |
| Serine hydroxymethyltransferase | 171232 | *Lactobacillus acidophilus* | Supports amino acid metabolism, essential for probiotic survival. |
| Ribose import ATP-binding protein (RbsA) | 193311 | *Lacticaseibacillus paracasei* | Regulates sugar transport, influencing gut microbiota composition. |
| Aspartate--tRNA ligase (aspS) | 74862 | *Lactiplantibacillus plantarum* | Aids in bacterial protein synthesis, supporting gut microbiota restoration. |
| Elongation factor Ts (tsf) | 81583 | *Lactobacillus helveticus* | Enhances bacterial translation efficiency, promoting gut microbiome stability. |
| Elongation factor Tu (tuf) | 60233 | *Bifidobacterium animalis subsp. lactis* | Supports microbial protein synthesis, aiding in gut microbiota restoration. |
| Glycine--tRNA ligase α-subunit (glyQ) | 140595 | *Lacticaseibacillus paracasei* | Essential for bacterial survival, maintaining probiotic function in the gut. |
| Peptide chain release factor 1 (prfA) | 17600 | *Bifidobacterium animalis subsp. lactis* | Regulates bacterial translation termination, maintaining microbial balance. |
| GMP reductase (guaC) | 73395 | *Lactobacillus acidophilus* | Involved in nucleotide metabolism, potentially supporting bacterial survival and gut barrier function. |
| Small ribosomal subunit protein uS13 (rpsM) | 221358 | *Streptococcus thermophilus* | Supports bacterial translation, contributing to probiotic stability. |
| DNA repair protein RecO (recO) | 240477 | *Lactobacillus helveticus* | Enhances bacterial survival in oxidative stress, supporting immune modulation. |
| Septation ring formation regulator EzrA (ezrA) | 38468 | *Lactiplantibacillus plantarum* | Maintains bacterial cell division, ensuring probiotic function. |
| 33 kDa chaperonin (hslO) | 28766 | *Lactobacillus acidophilus* | Aids in protein folding and stress response, improving probiotic survival under gut inflammation. |
| GTP cyclohydrolase 1 (folE) | 79482 | *Lactiplantibacillus plantarum* | Involved in folate biosynthesis, supporting bacterial metabolic activity and gut health. |
| Trigger factor (tig) | 33111 | *Lactobacillus acidophilus* | Assists in proper bacterial protein folding, ensuring gut microbiota stability. |
| Protein RecA (recA) | 46392 | *Bifidobacterium breve* | Enhances bacterial DNA repair, supporting probiotic survival in inflamed gut conditions. |
| Chaperone protein DnaK (dnaK) | 596424 | *Lactobacillus helveticus* | Provides stress resistance to probiotics, aiding in gut microbiota restoration. |
| Dihydroorotase (pyrC) | 66335 | *Streptococcus thermophilus* | Participates in nucleotide synthesis, ensuring bacterial replication and function. |
| Ribonuclease Z (rnz) | 335578 | *Lacticaseibacillus paracasei* | Regulates bacterial RNA processing, maintaining probiotic stability. |
| Tryptophan synthase β-chain (trpB) | 112911 | *Streptococcus thermophilus* | Supports tryptophan metabolism, which is crucial for immune modulation and gut barrier integrity. |
| MurG (murG) | 57757 | *Lactiplantibacillus plantarum* | Involved in bacterial cell wall synthesis, maintaining probiotic integrity. |
| Pup--protein ligase (pafA) | 336425 | *Bifidobacterium animalis subsp. lactis* | Regulates protein degradation in probiotics, ensuring bacterial homeostasis. |
| GTPase Der (der) | 309760 | *Lactiplantibacillus plantarum* | Facilitates bacterial ribosome assembly, contributing to microbiota stability. |
| Glucosamine-6-phosphate deaminase (nagB) | 72704 | *Lactobacillus helveticus* | Plays a role in carbohydrate metabolism, supporting probiotic growth. |
| Threonine--tRNA ligase (thrS) | 78472 | *Lactiplantibacillus plantarum* | Supports bacterial protein synthesis, essential for microbiota maintenance. |
| Serine--tRNA ligase (serS) | 100433 | *Bifidobacterium animalis subsp. lactis* | Essential for bacterial survival, aiding in microbiota restoration. |
| ATP-dependent 6-phosphofructokinase (pfkA) | 363233 | *Lactobacillus acidophilus* | Regulates energy metabolism in probiotics, enhancing their survival in the gut. |
| GMP synthase (guaA) | 498446 | *Lactobacillus acidophilus* | Plays a role in nucleotide synthesis, essential for probiotic DNA repair and growth. |
| 3-dehydroquinate synthase (aroB) | 139350 | *Streptococcus thermophilus* | Supports aromatic amino acid synthesis, crucial for gut microbiota metabolic balance. |
| ATP-dependent Clp protease (clpX) | 124439 | *Streptococcus thermophilus* | Maintains bacterial protein homeostasis, improving probiotic stress resistance. |
| Guanylate kinase (gmk) | 166079 | *Lactiplantibacillus plantarum* | Involved in nucleotide metabolism, supporting probiotic survival. |
| DNA-directed RNA polymerase α-subunit (rpoA) | 216044 | *Streptococcus thermophilus* | Facilitates bacterial gene expression, crucial for gut microbiota maintenance. |
| Elongation factor 4 (lepA) | 1261274 | *Bifidobacterium animalis subsp. lactis* | Regulates bacterial protein translation, enhancing probiotic efficiency. |
| Large ribosomal subunit protein uL1 (rplA) | 183989 | *Lactobacillus acidophilus* | Supports bacterial translation, crucial for microbiota stability. |
| Enolase 1 (eno1) | 1309050 | *Lactiplantibacillus plantarum* | Participates in glycolysis, ensuring probiotic metabolic function. |
| DNA ligase (ligA) | 74862 | *Lactiplantibacillus plantarum* | Aids in bacterial DNA repair, essential for probiotic stability in inflammatory conditions. |
| Neutral endopeptidase (pepO) | 11300 | *Lactobacillus helveticus* | May regulate peptide signaling in the gut, contributing to immune modulation. |
| Pyruvate oxidase (pox5) | 334718 | *Lactiplantibacillus plantarum* | Involved in metabolic pathways that support probiotic energy production. |
| ATP phosphoribosyltransferase (hisG) | 629405 | *Lacticaseibacillus paracasei* | Supports histidine biosynthesis, contributing to gut microbiota function. |
| Phenylalanine--tRNA ligase α-subunit (pheS) | 269738 | *Lactobacillus helveticus* | Essential for bacterial translation, maintaining probiotic homeostasis. |
| tRNA modification GTPase MnmE (mnmE) | 1341880 | *Lactobacillus helveticus* | Enhances bacterial survival and stress response, aiding probiotic persistence in the gut. |
| L-arabinose isomerase (araA) | 327375 | *Bifidobacterium animalis* | Supports carbohydrate metabolism, promoting beneficial microbiota growth. |
| 2,3-bisphosphoglycerate-dependent phosphoglycerate mutase (gpmA) | 250709 | *Streptococcus thermophilus* | Involved in glycolysis, improving energy metabolism for probiotic colonization. |
| NH3-dependent NAD(+) synthetase (nadE) | 274001 | *Lacticaseibacillus paracasei* | Supports cellular metabolism and oxidative stress resistance in probiotics. |
| Serine--tRNA ligase (serS) | 351135 | *Lacticaseibacillus paracasei* | Enhances protein biosynthesis in gut microbiota, aiding gut homeostasis. |
| Protein translocase subunit SecA (secA) | 1280985 | *Lactobacillus helveticus* | Assists in protein transport, enhancing probiotic functionality in the gut. |
| UvrABC system protein B (uvrB) | 826845 | *Streptococcus thermophilus* | Involved in DNA repair, promoting probiotic resilience in the inflamed gut. |
| DNA-directed RNA polymerase subunit beta' (rpoC) | 325231 | *Lactobacillus helveticus* | Regulates gene expression in probiotics, aiding adaptation in inflamed conditions. |
| ATP-dependent helicase/nuclease subunit A (addA) | 467789 | *Lactobacillus acidophilus* | Supports DNA repair in probiotics, maintaining their stability in the gut. |
| Endonuclease MutS2 (mutS2) | 248244 | *Lactobacillus helveticus* | Involved in DNA repair, promoting probiotic genomic stability. |
| DNA mismatch repair protein MutS (mutS) | 591650 | *Lactiplantibacillus plantarum* | Prevents mutations in probiotic bacteria, enhancing their resilience. |
| DNA polymerase IV (dinB) | 1381165 | *Streptococcus thermophilus* | Involved in stress-induced DNA repair, supporting probiotic survival. |
| Galactose-1-phosphate uridylyltransferase (galT) | 548059 | *Lactobacillus helveticus* | Important for carbohydrate metabolism, promoting probiotic function. |
| L-lactate dehydrogenase 1 (ldh1) | 962554 | *Lactobacillus acidophilus* | Produces lactate, supporting a healthy gut microbiome and pH balance. |
| tRNA uridine 5-carboxymethylaminomethyl modification enzyme MnmG (mnmG) | 652037 | *Lactiplantibacillus plantarum* | Enhances probiotic RNA modification and stress adaptation. |
| Alanine--tRNA ligase (alaS) | 1103588 | *Lacticaseibacillus paracasei* | Supports amino acid metabolism, promoting probiotic growth. |
| Aminopeptidase C (pepC) | 676779 | *Streptococcus thermophilus* | Aids in protein digestion, improving nutrient availability for mucosal healing. |
| Enolase (eno) | 342128 | *Streptococcus thermophilus* | Involved in glycolysis, enhancing probiotic metabolism. |
| Chaperone protein DnaK (dnaK) | 363273 | *Bifidobacterium animalis* | Assists in protein folding and stress response, promoting probiotic viability. |
| CRISPR-associated endonuclease Cas9 (cas9-1, cas9-2) | 1737792 | *Streptococcus thermophilus* | Potential for genome editing in probiotics, modulating microbiota for IBD therapy. |
| S-layer protein (slpA) | 1336567 | *Lactobacillus acidophilus* | Aids in bacterial adhesion to gut epithelium, supporting mucosal protection. |
| S-ribosylhomocysteine lyase (luxS) | 86853 | *Lactiplantibacillus plantarum* | Quorum sensing, bacterial communication  Microbiota restoration |
| ATP phosphoribosyltransferase (hisZ) | 23556 | *Lactiplantibacillus plantarum* | Amino acid biosynthesis  Supports bacterial metabolism |
| Acetylornithine aminotransferase (argD) | 58803 | *Lactiplantibacillus plantarum* | Arginine metabolism  Mucosal healing & tissue repair |
| Serine--tRNA ligase 1 (serS1) | 16268 | *Lactiplantibacillus plantarum* | Protein biosynthesis  General bacterial function |
| Isopentenyl-diphosphate isomerase (fni) | 20198 | *Lactiplantibacillus plantarum* | Isoprenoid biosynthesis  Bacterial viability |
| Ribosomal RNA methyltransferase H (rlmH) | 85067 | *Lactiplantibacillus plantarum* | Ribosomal function  Supports protein synthesis |
| Bile salt hydrolase/transferase (cbh) | 53940 | *Lactiplantibacillus plantarum* | Bile metabolism  Microbiota restoration |
| ATP synthase subunit alpha (atpA) | 292576 | *Lactiplantibacillus plantarum* | Energy metabolism  Supports probiotic function |
| DNA mismatch repair protein (mutL) | 467718 | *Lactiplantibacillus plantarum* | DNA repair  Maintains probiotic viability |
| Phenylalanine--tRNA ligase (pheT) | 427842 | *Lactiplantibacillus plantarum* | Protein biosynthesis  General bacterial function |
| Sulfate adenylyltransferase (sat) | 162685 | *Lactiplantibacillus plantarum* | Sulfur metabolism  Microbial interactions |
| Elongation factor P 1 (efp1) | 36599 | *Lactobacillus acidophilus* | Enhances translation  Supports probiotic protein expression |
| Large ribosomal subunit protein uL11 (rplK) | 54352 | *Lactobacillus acidophilus* | Ribosomal structure  Aids bacterial protein synthesis |
| HPr kinase/phosphorylase (hprK) | 18386 | *Lactobacillus acidophilus* | Sugar metabolism regulation  Microbiota colonization |
| Proline--tRNA ligase (proS) | 351844 | *Lactobacillus acidophilus* | Protein synthesis  General bacterial function |
| Formate--tetrahydrofolate ligase 1 (fhs1) | 164927 | *Lactobacillus acidophilus* | One-carbon metabolism  Energy balance |
| Ribonuclease HII (rnhB) | 538797 | *Lactobacillus acidophilus* | DNA repair  Bacterial survival |
| Ribose-5-phosphate isomerase A (rpiA) | 23617 | *Lactobacillus helveticus* | Central metabolism  Bacterial viability |
| Cysteine--tRNA ligase (cysS) | 23204 | *Lactobacillus helveticus* | Protein biosynthesis  General bacterial function |
| SsrA-binding protein (smpB) | 29947 | *Lactobacillus helveticus* | Ribosomal rescue  Bacterial adaptation |
| GTPase Era (era) | 536749 | *Lactobacillus helveticus* | Ribosome biogenesis  Supports bacterial function |
| Peptide deformylase (def) | 38501 | *Lactobacillus helveticus* | Protein maturation  Bacterial survival |
| tRNA sulfurtransferase (thiI) | 381158 | *Lactobacillus helveticus* | Sulfur metabolism  Probiotic function |
| D-alanine--D-alanine ligase (ddl) | 85980 | *Lactobacillus helveticus* | Peptidoglycan biosynthesis  Supports bacterial cell wall integrity |
| ATP-dependent 6-phosphofructokinase (pfkA) | 390947 | *Lactobacillus helveticus* | Glycolysis regulation  Supports probiotic survival |
| Ribosome-binding factor A (rbfA) | 34278 | *Lacticaseibacillus paracasei* | Ribosomal biogenesis  Aids bacterial adaptation |
| D-alanine--D-alanine ligase (ddl) | 126245 | *Lacticaseibacillus paracasei* | Peptidoglycan synthesis  Strengthens bacterial cell wall |
| Acetate kinase (ackA) | 26775 | *Lacticaseibacillus paracasei* | Acetate metabolism  SCFA production & gut microbiota support |
| Competence-damage inducible protein (cinA) | 25537 | *Lacticaseibacillus paracasei* | Stress response  Enhances probiotic resistance |
| Phosphoglycerate kinase (pgk) | 338751 | *Lacticaseibacillus paracasei* | Glycolysis enzyme  Supports bacterial survival |
| Teichoic acid D-alanyltransferase (dltB) | 151537 | *Streptococcus thermophilus* | Cell wall modification  Enhances probiotic stability |
| UDP-glucose 4-epimerase (galE) | 171810 | *Streptococcus thermophilus* | Carbohydrate metabolism  Supports bacterial energy supply |
| Spermidine/putrescine transporter (potA) | 22818 | *Streptococcus thermophilus* | Polyamine transport  Enhances gut barrier function |
| CRISPR system Cms protein (csm5) | 21809 | *Streptococcus thermophilus* | Bacterial immunity  Probiotic stability |
| 3-isopropylmalate dehydratase (leuD) | 24467 | *Streptococcus thermophilus* | Leucine biosynthesis  Supports bacterial growth |
| Urease subunit alpha (ureC) | 210112 | *Streptococcus thermophilus* | Urease activity  Impacts nitrogen metabolism |
| Beta-galactosidase (lacZ) | 656773 | *Streptococcus thermophilus* | Lactose metabolism  Beneficial for gut microbiota |
| Methionine--tRNA ligase (metG) | 29821 | *Bifidobacterium animalis subsp. lactis* | Protein biosynthesis  Ensures probiotic survival |
| Bifunctional protein GlmU (glmU) | 17057 | *Bifidobacterium animalis subsp. lactis* | Cell wall synthesis  Maintains bacterial integrity |
| Large ribosomal subunit protein bL12 (rplL) | 6196 | *Bifidobacterium animalis subsp. lactis* | Protein translation  Supports bacterial function |
| Protein NrdI (nrdI) | 53286 | *Bifidobacterium animalis subsp. lactis* | Ribonucleotide metabolism  Affects DNA synthesis |
| ATP synthase subunit alpha (atpA) | 34284 | *Bifidobacterium animalis subsp. lactis* | ATP production  Crucial for probiotic survival |
| Exo-alpha-(1->6)-L-arabinopyranosidase (HMPREF9228_1477) | 32264 | *Bifidobacterium breve* | Carbohydrate metabolism  Supports prebiotic activity & microbiota restoration |
